# *In vitro* probiotic characteristics and whole-genome sequencing analysis of porcine-derived lactic acid bacteria

**DOI:** 10.7717/peerj.21496

**Published:** 2026-07-15

**Authors:** Shumei Li, Heming Zhang, Xin Xing, Yuyang Wang, Ying Shao, Zhao Qi

**Affiliations:** 1 Anhui Province Key Laboratory of Smart Agricultural Technology and Equipment, Hefei, China; 2 School of Information and Artificial Intelligence, Anhui Agricultural University, Hefei, Anhui Province, China; 3 Anhui Provincial Key Laboratory of Veterinary Pathobiology and Disease Control, Hefei, China

**Keywords:** Lactic acid bacteria, Probiotic biosafety, Horizontal gene transfer, Genomics, Mobile genetic elements

## Abstract

Lactic acid bacteria (LAB) have potential applications as probiotics, but the antibiotic resistance genes (ARGs) and virulence factor genes (VFGs) they carry pose significant public health risks. In this study, eight strains of LAB were isolated from fecal samples of large-scale pig farms, and their probiotic potential and safety were systematically evaluated through in vitro functional assays and whole-genome sequencing. The results showed that *Pediococcus pentosaceus* R124 exhibited high adhesion to intestinal epithelial cells (54.11%) and strong coaggregation ability (34.9%). *Lactiplantibacillus plantarum* Z108 demonstrated relatively strong acid resistance (24.18% survival rate at pH 2.0), while *Enterococcus faecium* F130 showed the highest tolerance to high concentrations of bile salts (6.28%). All strains exhibited a non-hemolytic γ -hemolysis phenotype, but antibiotic susceptibility testing revealed widespread phenotypic multidrug resistance. Interestingly, while *P. pentosaceus* R124 and *L. plantarum* Z108 showed resistance to certain antibiotics, genomic analysis indicated they harbored no acquired ARGs or VFGs, suggesting that their observed resistance may stem from intrinsic mechanisms. *E. faecium* F130 harbored four VFGs and thirteen ARGs, eight of which were located on plasmids. Importantly, the filter-mating experiment indicated that the antibiotic-resistant plasmid PlasmidE and its associated resistance gene *ANT(4’)-Ib* from *E. faecium* F130 could be horizontally transferred to recipient strains, conferring antibiotic resistance. This study suggests that, while all isolated strains possess certain probiotic potential, their safety varies according to species and strain-specific characteristics. *P. pentosaceus* R124 and *L. plantarum* Z108 are promising candidates with both functional and genetic safety, while the risk of resistance spread from *E. faecium* F130 highlights the necessity of whole-genome safety assessments in probiotic screening.

## Introduction

Antibiotic resistance has become a major global public health threat (Ali et al., 2022; Banerji et al., 2019; Lessa & Sievert, 2023). A report in 2022 indicated that more than six million deaths were directly or indirectly caused by antibiotic resistance in 2019 (Murray et al., 2022). In response to this crisis, China’s Ministry of Agriculture and Rural Affairs issued Announcement No. 194 in 2019, banning the use of antibiotics in animal feed, thereby prompting the livestock industry to seek safer alternatives.

Lactic acid bacteria (LAB), a group of Gram-positive bacteria, have been widely used as alternatives to antibiotics due to their probiotic properties (Abedin et al., 2024). They enhance feed conversion rates and inhibit pathogen colonization by producing organic acids and bacteriocins, mechanisms that have been extensively confirmed in both basic and recent research (Adetoye et al., 2018; Kavanova et al., 2025; Peng et al., 2025; Wang et al., 2024). Furthermore, LAB strains isolated from breast milk and infant feces exhibit significant immunomodulatory effects and are effective against diarrheal pathogens. Some of these strains remain sensitive to antibiotics, making them promising candidates for probiotics (Obisesan, Abiodun & Ayeni, 2024).

Although LAB plays an important role in promoting health, recent studies have revealed their potential as reservoirs of antibiotic resistance genes (ARGs), which could exacerbate the spread of antibiotic resistance, a risk that should not be overlooked. Research has shown that LAB from different sources exhibit varying levels of antibiotic resistance, particularly to key antibiotic classes, with several resistance genes (such as *parC*, *aac(6′)Ii*, *ermB*, and *tetM*) confirmed in LAB (Duche et al., 2023). The spread of these resistance genes not only affects LAB itself but may also impact other bacterial populations through horizontal gene transfer (HGT) (Rodrigo-Torres et al., 2019; Stefańska et al., 2021). For example, the *Enterococcus* genus is a common vector for antibiotic resistance, particularly in animal sources, where resistance to last-resort antibiotics, such as vancomycin, has been reported. These phenomena underscore the direct threat that antibiotic resistance poses to public health (Aqib & Alsayeqh, 2022; Caddey et al., 2025). This risk is not limited to ARGs. *Enterococcus* strains from dairy products may also carry multiple resistance and virulence determinants (Shanab et al., 2025). Livestock environments, characterized by high antimicrobial consumption, have emerged as primary reservoirs for ARGs and virulence factor genes (VFGs) (Liu et al., 2025). Metagenomic analysis shows over 11,250 HGT events in the “pig-environment-human” transmission network, with more than 50% of ARGs mediated by plasmids (Wang et al., 2025b). Additionally, the application of manure accelerates the penetration of high-risk genes into the soil and host microbiomes (Pan et al., 2024). In this high-pressure ecological niche, LAB frequently builds potential pathways for resistance transmission through mobile genetic element (MGEs), which could spread to humans *via* the food chain. Among various LAB strains, including those from traditional foods, key risk genes for chloramphenicol, vancomycin, sulfonamides, and aminoglycosides have been identified (Berreta, Baumgardner & Kopper, 2020; Li et al., 2019; Zarzecka, Chajecka-Wierzchowska & Zadernowska, 2022). Recent studies have confirmed these risks, such as the emergence of colistin resistance in livestock LAB strains, and broad-spectrum resistance exhibited by strains from dairy products and aquaculture (Ray, Ashwini & Halami, 2024; Yilmaz et al., 2025). The horizontal transfer of these resistance genes can disrupt gut microbiota balance, impair intestinal barrier function, and affect host immune responses, further emphasizing the urgency of establishing more stringent safety assessment frameworks for LAB (Forster et al., 2022; Rozman et al., 2023; Tóth et al., 2023; Yang & Yu, 2019). Despite existing studies revealing the potential risks of LAB in the spread of antibiotic resistance, systematic investigations of specific ecological niches, such as pig farm strains, remain insufficient (Colautti et al., 2022; Partridge et al., 2018). Furthermore, although the role of MGEs in the spread of antibiotic resistance is recognized, current safety assessments typically rely on bioinformatic predictions or *in vitro* antibiotic resistance phenotype analysis, lacking experimental validation of the transferability of resistance genes (Bányai, 2025; McInnes et al., 2020). Therefore, this study selected porcine-derived lactic acid bacteria isolates as the research subjects. By integrating phenotypic assays, whole-genome sequencing, and conjugation experiments, we aimed to identify promising probiotic candidates while empirically validating the horizontal transfer potential of high-risk resistance determinants. This comprehensive approach provides critical insights into both the probiotic application and the public health security of LAB in livestock environments.

## Materials & Methods

### Sampling

Fecal samples were collected from a large-scale pig farm in Anhui Province, China. The samples were collected in sterile zipper bags, sealed, and transported to the laboratory *via* cold chain transport in an ice box (4 °C). The recipient strain for the conjugation experiment, *Enterococcus faecalis* JH2-2, was kindly provided by Ms. Xinxin Shan from the College of Animal Science and Technology, Anhui Agricultural University. The indicator strains (*Staphylococcus aureus* ATCC 25923 and *Escherichia coli* ATCC 25922) and the porcine intestinal epithelial cell line IPEC-J2 were maintained by the Key Laboratory of Veterinary Pathology and Disease Prevention of Anhui Province.

### Strain isolation and screening of lactic acid bacteria

According to the established protocol (AlKalbani, Turner & Ayyash, 2019), one gram of each sample was weighed and subjected to 10-fold serial dilutions in sterile Phosphate-Buffered Saline (PBS). Three dilutions, 10^−6^, 10^−7^, and 10^−8^, were prepared. One hundred microliters of each dilution were evenly spread onto MRS agar plates (HB0384-5, Qingdao Hope Bio-Technology Co., Ltd., Qingdao, China) supplemented with 1% CaCO_3_. The plates were then transferred to an anaerobic glove box (Coy Laboratory Products), where an oxygen-free atmosphere (typically maintained with a gas mixture of 5% H_2_, 5% CO_2_, and 90% N_2_) was established and strictly monitored. The plates were incubated within the integrated incubator of the glove box at 37 °C for 24 h. Colonies exhibiting a calcium-dissolving halo were selected, purified, and subjected to Gram staining (Beveridge, 2001). Only Gram-positive strains with rod or coccal morphology were selected for subsequent identification. Isolates were stored at −80 °C in 20% (v/v) glycerol.

To identify the isolates, Polymerase Chain Reaction (PCR) amplification was performed using primers 27-F (5′-AGAGTTTGATCCTGGCTCAG-3′) and 1492R (5′-TACGGCTACCTTGTACGACTT-3′) under the following conditions: an initial denaturation at 95 °C for 3 min; followed by 35 cycles of denaturation at 95 °C for 15 s, annealing at 52.4 °C for 15 s, and extension at 72 °C for 45 s; and a final extension at 72 °C for 5 min. The amplification products were verified by 1% agarose gel electrophoresis at 110 V for 30 min, yielding a target band of approximately 1,500 bp. Qualified samples were sent to Nanjing Qingke Biotechnology Co., Ltd. for Sanger sequencing, and species identification was completed by BLAST comparison against the NCBI database (similarity >99%).

### Safety evaluation

#### Hemolytic activity

The assessment of hemolytic activity was conducted following the protocol established by Wang et al. (2025a) and Wang et al. (2025b). The selected strains were cultured overnight in MRS broth at 37 °C. The culture was then streaked onto blood agar plates (Changde Bickman Bio) and incubated at 37 °C for 24 h. Hemolytic activity was determined based on the following criteria: α-hemolysis, characterized by a green discoloration around the colonies (partial hemolysis); β-hemolysis, shown by a clear halo surrounding the colonies (complete hemolysis); and γ-hemolysis, where no visible hemolysis is observed (non-hemolytic).

#### Antibiotic resistance analysis

Antibiotic susceptibility was determined using the disk diffusion method (Kirby-Bauer, K-B method) (Bauer et al., 1966) on Mueller-Hinton agar. Based on the relevance to both veterinary and human medicine and their diverse mechanisms of action (Collado et al., 2007; Xie et al., 2023), this study assessed susceptibility to the following fourteen antibiotics: Penicillin (10 IU), Ampicillin (10 µg), Amoxicillin-Clavulanic Acid (20/10 µg), Cefradine (30 µg), Cefazolin (30 µg), Cefotaxime (30 µg), Gentamicin (10 µg), Kanamycin (30 µg), Streptomycin (10 µg), Clarithromycin (15 µg), Erythromycin (15 µg), Sulfisoxazole (250 µg), Trimethoprim-Sulfamethoxazole (1.25/23.75 µg), and Florfenicol (30 µg). The plates were incubated at 37 °C for 18 h, with *S. aureus* serving as the quality control strain. The diameter of the inhibition zones was measured using a caliper, and the results were interpreted according to the Clinical and Laboratory Standards Institute (CLSI) M100 guidelines (2021) breakpoints for *Enterococcus* spp. (Ganapathiwar & Bhukya, 2023). In this study, multidrug resistance was defined as resistance to at least one antibiotic in three or more classes of antibiotics.

### Evaluating probiotic properties

#### Growth and acid production assessment

The activated bacterial strains were inoculated into MRS broth (1% v/v), with the initial inoculum standardized to an OD600 of approximately 1.0. Inoculated cultures were incubated at 37 °C for 24 h, using uninoculated MRS broth as a negative control. The OD600 value and pH value were measured and recorded every two h using a spectrophotometer and a digital pH meter, respectively.

#### Acid, bile salt, and temperature tolerance

The activated LAB cultures were standardized to an initial inoculum of approximately 10^8^ CFU/mL and then subjected to stress treatments. For acid tolerance, the strains were inoculated into MRS broth adjusted to pH 2.0, pH 3.0, and pH 5.0, and incubated at 37 °C for 4 h. MRS broth with no pH adjustment (approximately 6.5) was used as a control. For bile salt tolerance, the strains were tested under the same incubation conditions (37 °C for 4 h) using MRS broth containing 0.1%, 0.3%, and 0.5% (w/v) porcine bile salts. For thermal tolerance, strains were exposed to 40 °C, 50 °C, and 60 °C for 5 min and 10 min, respectively. Following all stress treatments, samples were serially diluted and plated onto MRS agar for counting to determine the survival rate of the strains. Survival rate was calculated by the plate counting method:

\begin{eqnarray*}Survival~(\%)= \frac{{S}_{2}}{{S}_{1}} \times 100\% \end{eqnarray*}

where S_1_ represents the number of viable cells CFU/mL after 4 h of incubation in MRS broth adjusted to pH 2.0, pH 3.0, and pH 5.0, respectively, and S_2_ represents the number of viable cells CFU/mL after 4 h of incubation in the unadjusted pH control MRS broth.

#### Bacterial coaggregation assay

The co-aggregation ability of the LAB isolates with the pathogenic indicator strains, *S. aureus* ATCC 25923 and *E. coli* ATCC 25922, was assessed according to a previously described method (Li et al., 2020). Activated LAB and pathogenic bacteria were centrifuged at 5,000 × g for 15 min, washed, and resuspended in PBS, with the final cell suspensions adjusted to an OD600 of 0.8 ± 0.02 (approximately 10^8^ CFU/mL). Equal volumes (two mL) of the LAB suspension and the pathogenic bacterial suspension were mixed and allowed to stand undisturbed at room temperature for 5 h. A four mL suspension of the single bacterial strain served as the control group. The OD600 of all suspensions was measured. The co-aggregation rate was calculated using the following formula:

\begin{eqnarray*}Co-aggregation~(\%)= \frac{ \frac{{C}_{x}+{C}_{y}}{2} -{C}_{(x+y)}}{({C}_{x}+{C}_{y})\times 100\%} \end{eqnarray*}

where C_*x*_ and C_*y*_ represent the absorbances of the two bacterial suspensions, and C_(__*x*__+__*y*__)_ represents the absorbance of the mixed bacterial suspension.

#### Cell surface hydrophobicity

Cell surface hydrophobicity was measured using the xylene-water two-phase separation method (Xu et al., 2009). The activated LAB cells were washed with PBS, and the cell suspension was adjusted to an OD600 of 0.80 ± 0.02. Three milliliters of the bacterial suspension were mixed with one mL of xylene and vortexed for 30 s. The mixture was allowed to stand undisturbed at room temperature for 15 min to allow phase separation. After removing the organic phase, the OD600 of the aqueous phase was measured. Surface hydrophobicity (%) was calculated using the following formula:

\begin{eqnarray*}Hydrophobicity~(\%)= \frac{{H}_{0}-{H}_{t}}{{H}_{0}} \times 100\% \end{eqnarray*}

where H_0_ is the absorbance at OD600 of the mixed bacterial suspension after vortexing, and H_t_ is the absorbance at OD600 of the aqueous phase after 15 min of static incubation.

#### Adhesion ability

Adhesion ability was determined following the method described by Li et al. (2020). IPEC-J2 cells were seeded into 24-well plates and cultured until 80% to 90% confluence. The FITC-labeled LAB cell suspension (10^8^ CFU/mL) was co-incubated with IPEC-J2 cells at 37 °C with 5% CO_2_ for 1 h. The cells were then washed with PBS and subjected to trypsin digestion. The fluorescence intensity was measured at Ex/Em = 485 nm/530 nm. The adhesion rate was calculated using the following formula:

\begin{eqnarray*}Adhesion~(\%)= \frac{{A}_{1}-{A}_{0}}{{A}_{2}-{A}_{0}} \times 100\% \end{eqnarray*}

where A_0_ is the fluorescence value of the blank control, A_1_ is the fluorescence value of the adhered bacteria, and A_2_ is the total fluorescence value of the added bacteria.

#### Biochemical identification

For biochemical identification, activated strains were suspended in physiological saline, and the turbidity was adjusted to 0.5 McFarland standards. One hundred microliters (100 µL) of the bacterial suspension were inoculated into LAB biochemical identification tubes (9 types, GB4789 standard, product number 21318, purchased from Qingdao Nissui Biotechnology). The identification tubes were incubated at 36 °C for 2–7 days, and the biochemical reactions were observed and recorded.

### Whole-genome sequencing and analysis

#### Genome sequencing and bioinformatics analysis

Genomic DNA was extracted from stationary-phase bacterial cultures (anaerobically cultured in MRS medium at 37 °C) using the Sangon^®^ Bacterial Genomic DNA Extraction Kit (Cat. No.: B518225, Sangon Biotech (Shanghai) Co., Ltd.). Subsequently, a hybrid sequencing strategy combining PacBio RS II Single-Molecule Real-Time (SMRT) sequencing and Illumina sequencing was employed for genome sequencing (Majorbio, Shanghai, China). Coding sequences (CDS) were predicted using Prodigal (V2.6.3) (Hyatt et al., 2010), and plasmid genomes were predicted using GeneMark.hmm (v2.0) (Besemer & Borodovsky, 2005). Genomic islands were predicted using IslandViewer4 (Bertelli et al., 2017), prophages using Phigaro (Starikova et al., 2020) (v2.4.0), and CRISPR clusters within the genome using CRISPRCasFinder (Couvin et al., 2018) (v4.2.20). Transposons and insertion sequences were predicted using ISEScan (Xie & Tang, 2017) (v1.7.2) and TransposonPSI (Haas, 2007) (v1.0), respectively. Functional annotation of the predicted CDS was performed through comparative analysis against specialized databases, including the Carbohydrate-Active enzymes database (CAZy), the Virulence Factor Database (VFDB), and the Antibiotic Resistance Genes Database (ARDB).

#### Comparative pan-genome analysis

The full genome sequencing data for all bacteria available in the NCBI Genomes database were downloaded and screened. For *Lactiplantibacillus plantarum* (formerly *Lactobacillus plantarum*) and *Enterococcus faecium*, the screening criteria were set for strains with genome sequences uploaded between 2021 and 2024 that were assembled to a complete level. Due to the limited data for *Pentosacoccus pentosaceus*, the screening criterion for this species was broadened to include all genome sequences uploaded between 2021 and 2024. The pan-genome for each species was constructed using *Roary* (v3.7.0), with a protein identity threshold set at 90%. Genes present in ≥ 99% of the strains were defined as core genes. The *mobileOG-db* database (Brown et al., 2022) was used to predict the MGEs present in the pan-genome. *Plasmidfinder* (Carattoli et al., 2014) was employed to predict plasmids in the pan-genome. ARGs and VFGs were identified using *ABRicate* (v1.0.1), cross-referenced with the Comprehensive Antibiotic Resistance Database (CARD) and VFDB databases, respectively.

### Horizontal gene transfer experiment

The transferability of antimicrobial resistance was evaluated using the membrane filter mating method (Ojha, Shah & Mishra, 2021). For the conjugation experiment, *Enterococcus faecium* F130 (donor strain, kanamycin-resistant and rifampicin-sensitive) and *E. faecalis* JH2-2 (recipient strain, rifampicin-resistant and kanamycin-sensitive) were cultured to the mid-logarithmic phase, reaching an OD600 of approximately 0.6. The donor and recipient cultures were then mixed at a 1:4 volume ratio (200 µL:800 µL). The mixture was collected by filtration through a 0.45 µm membrane filter, which was then placed onto an antibiotic-free MRS agar plate and incubated at 37 °C for 20 h to facilitate conjugation. The donor and recipient were processed separately as controls. Following conjugation, the cell suspensions were serially diluted and plated onto MRS agar containing both kanamycin (50 µg/mL) and rifampicin (50 µg/mL) for the selective isolation of transconjugants. Transconjugant colonies were verified for the presence of the transferred plasmid by16S rRNA PCR amplification (using specific primers listed in Table 1). The PCR system was identical to that described in ‘Strain isolation and screening of lactic acid bacteria’.

### Statistical analysis

All results are expressed as the mean of three independent experiments ± standard deviation. Statistical analysis was performed using SPSS software (version 22.0; IBM Corp., Armonk, NY, USA). The normality of data distribution and the homogeneity of variances were assessed using the Shapiro–Wilk test and Levene’s test, respectively, prior to one-way analysis of variance (ANOVA). One-way ANOVA was employed, followed by Tukey’s *post-hoc* test for pairwise comparisons. Throughout the manuscript, the different lowercase letters (*e.g.*, a, b, c, d) presented in the figures indicate statistically significant differences among the eight isolates within the same experimental group at the *p* < 0.05 level. Differences were considered statistically significant when the *p*-value was less than 0.05. All statistical comparisons were performed using two-tailed tests.

## Results

### Isolation and identification of lactic acid bacteria

**Figure 1:**
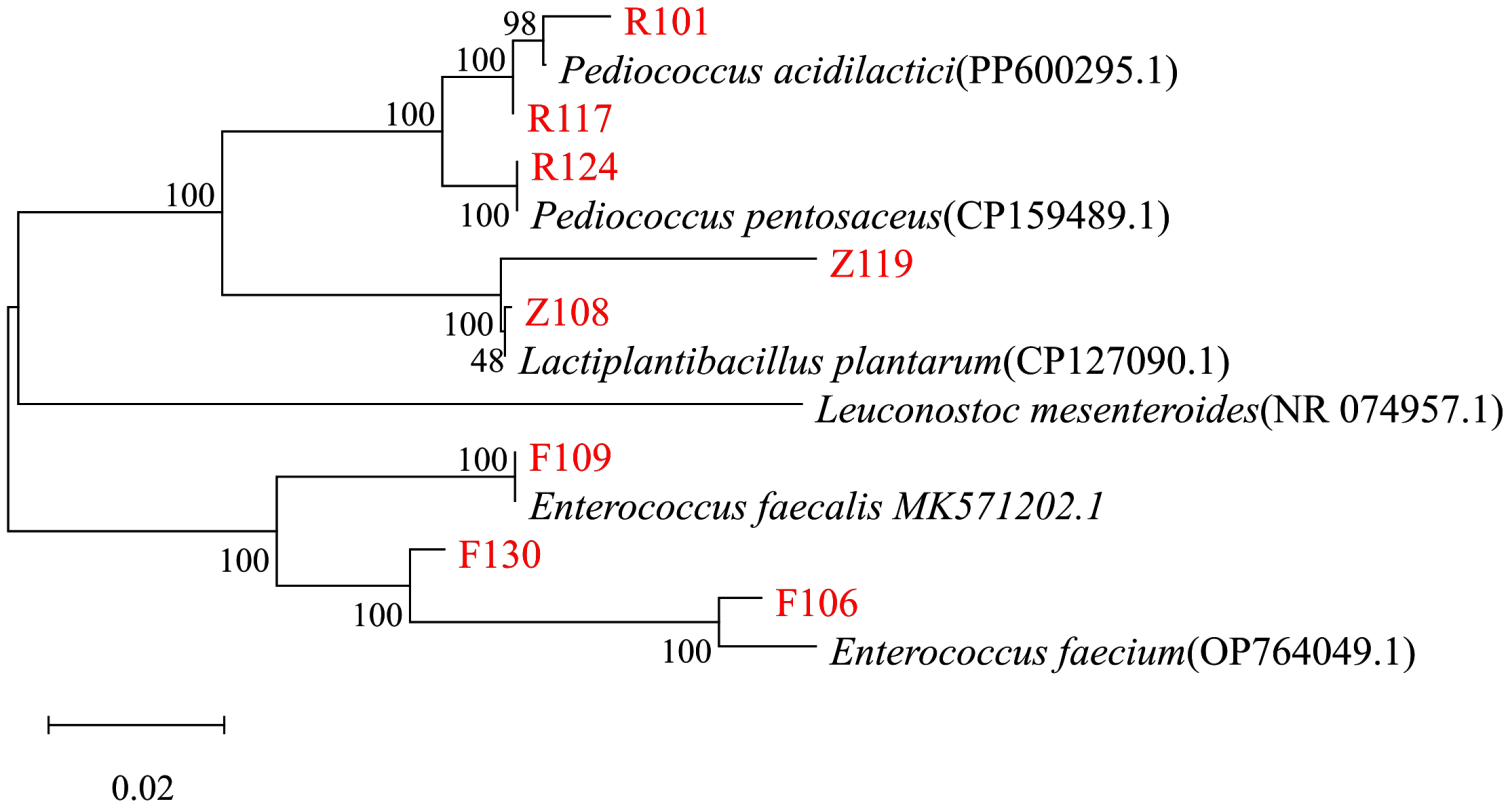
Phylogenetic tree of eight isolated lactic acid bacteria strains based on 16S rRNA gene sequences.

In this study, eight strains of LAB were isolated and initially identified through 16S rRNA gene sequencing. These strains were classified into three genera: *Enterococcus* (three strains), *Pediococcus* (three strains), and *Lactiplantibacillus* (two strains). The identified species included *Enterococcus faecium* (F106, F130), *Enterococcus faecalis* (F109), *Pediococcus acidilactici* (R101, R117), *Pediococcus pentosaceus* (R124), and *Lactiplantibacillus plantarum* (Z108, Z119). Based on the 16S rRNA sequencing results, a phylogenetic tree was constructed using the Neighbor-Joining (N-J) method in MEGA software (version 12.0; Pennsylvania State University, PA, USA) with 1,000 bootstrap replicates (Fig. 1).

**Table 1:** Primers used for strain identification and plasmid transfer validation in this study. The annealing temperature for all primer pairs was optimized at 52.4 °C.

**Primer name**	**Primer sequence (5′–3′)**	**Amplicon size (bp)**
***E. faecium*** **-F**	TTGAGGCAGACCAGATTGACG	658
***E. faecium*** **-R**	TATGACAGCGACTCCGATTCC	
***E. faecalis*** **-F**	ACTTATGTGACTAACTTAACC	360
***E. faecalis*** **-R**	TAATGGTGAATCTTGGTTTGG	
**PlasmidE-F**	ATGGCTGAAAGTAAGAAACGAGTGT	143
**PlasmidE-R**	TCTGCTTCCTTTCTTGCATATTCCT	

**Notes.**

The annealing temperature for all primer pairs was optimized at 52.4 °C.

### Safety assessment of lactic acid bacteria

Hemolytic activity is a key indicator in evaluating the safety of probiotics. In this study, hemolysis tests were conducted on the eight isolated strains, and the results showed that all strains exhibited a γ-hemolytic phenotype (*i.e.,* no hemolysis), with no clear or green zones indicating erythrocyte breakdown observed on blood agar plates (see Figs. S1–S8). This indicates that these strains do not possess the ability to lyse host red blood cells, demonstrating good phenotypic safety.

Furthermore, the antibiotic susceptibility of the eight LAB strains was evaluated, and the results were interpreted as Susceptible (S), Intermediate (I), or Resistant (R) according to the CLSI M100 (2021) guidelines (Table 2). The testing revealed widespread multidrug resistance among the isolates. The strains were generally sensitive to β-lactam antibiotics, but displayed relatively high resistance rates to aminoglycosides. Specifically, the resistance rates to gentamicin and kanamycin were 87.5% (7/8 strains) and 100%, respectively. Notably, the *Enterococcus* strains (F106, F109, F130) exhibited significantly higher resistance levels and a broader spectrum of resistance.

**Table 2:** Antibiotic susceptibility of the isolated lactic acid bacteria strains. The following are the abbreviations for antibiotics: FON (Florfenicol), SXT (TrimethoprimSulfamethoxazole), SIZ (Sulfisoxazole), ERY (Erythromycin), CLR (Clarithromycin), SM (Streptomycin), Kana (Kanamycin), GM (Gentamicin), CTX (Cefotaxime), CEZ (Cefazolin), RAD (Cefradine), AMC (Amoxicillin-Clavulanic Acid), AMP (Ampicillin), and PCN (Penicillin). Antibiotic susceptibility results (S: Susceptible, I: Intermediate, R: Resistant) were interpreted according to the CLSI M100 (2021) guidelines breakpoints for *Enterococcus* spp.

**Antibiotics**	Susceptibility							
	F106	F130	F109	R101	R117	R124	Z108	Z119
FON	S	S	R	I	S	R	I	S
SXT	R	R	S	S	R	R	S	R
SIZ	R	R	R	S	R	R	S	R
ERY	R	R	R	S	S	R	S	R
CLR	R	I	R	I	R	S	I	S
SM	R	R	R	R	R	I	R	R
Kana	R	R	R	R	R	R	R	R
GM	R	R	R	R	R	R	R	R
CTX	I	R	I	S	I	S	I	S
CEZ	I	I	I	I	S	I	I	I
RAD	S	S	I	S	S	S	I	S
AMC	I	I	S	S	S	I	R	R
AMP	R	R	S	S	S	R	R	I
PCN	I	S	R	R	R	S	I	S

**Notes.**

FON Florfenicol SXT Trimethoprim-Sulfamethoxazole SIZ Sulfisoxazole ERY Erythromycin CLR Clarithromycin SM Streptomycin Kana Kanamycin GM Gentamicin CTX Cefotaxime CEZ Cefazolin RAD Cefradine AMC Amoxicillin-Clavulanic Acid AMP Ampicillin PCN Penicillin

Antibiotic susceptibility results (S: Susceptible, I: Intermediate, R: Resistant) were interpreted according to the CLSI M100 (2021) guidelines breakpoints for *Enterococcus* spp.

### Evaluation of probiotic properties of lactic acid bacteria

#### Growth and acidification kinetics

**Figure 2:**
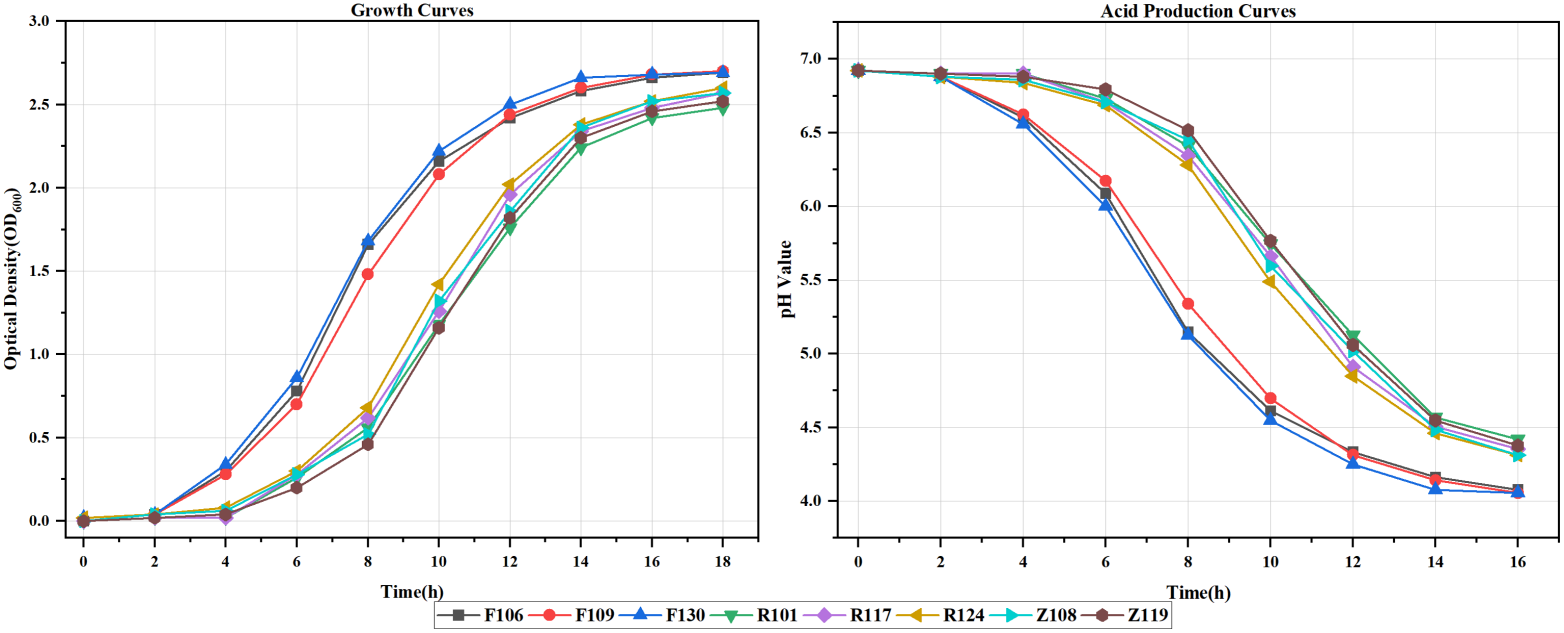
Growth and acidification curves of the isolated lactic acid bacteria strains. Growth curves were monitored by measuring the optical density at 600 nm (OD600). Acidification curves were determined by measuring the pH changes in the culture supernatant.

Strains from different genera exhibited distinct growth patterns (Fig. 2). *Enterococcus* strains (F106, F109, F130) grew more rapidly, entering the logarithmic growth phase at 4 h and reaching the stationary phase at 14 h. In contrast, *Pediococcus* strains (R101, R117) and *Lactobacillus* strains (Z108, Z119) had longer lag phases, requiring 16 h to reach the stationary phase. This growth difference was also reflected in the acidification patterns. The *Enterococcus* strains rapidly lowered the pH to approximately 4.0 within 14 h, while strains from other genera took 16 h to reach a final pH of around 4.3.

#### Stress tolerance

**Figure 3:**
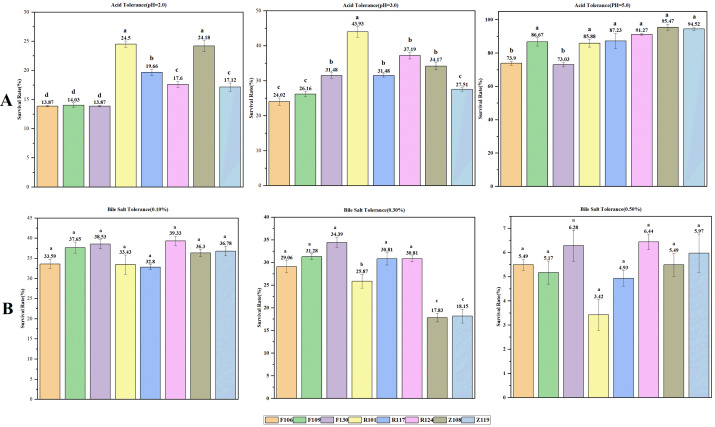
Evaluation of acid and bile salt tolerance of the isolated lactic acid bacteria strains. (A) Acid tolerance (pH 2.0−5.0) after 3 h of treatment. (B) Bile salt tolerance (0.1%−0.5% porcine bile salts) after 4 h of treatment. The different lowercase letters (*e.g.*, a, b, c, d) presented in the figures indicate statistically significant differences among the eight isolates within the same experimental group at the *p* < 0.05 level. Differences were considered statistically significant when the *p*-value was less than 0.05.

**Figure 4:**
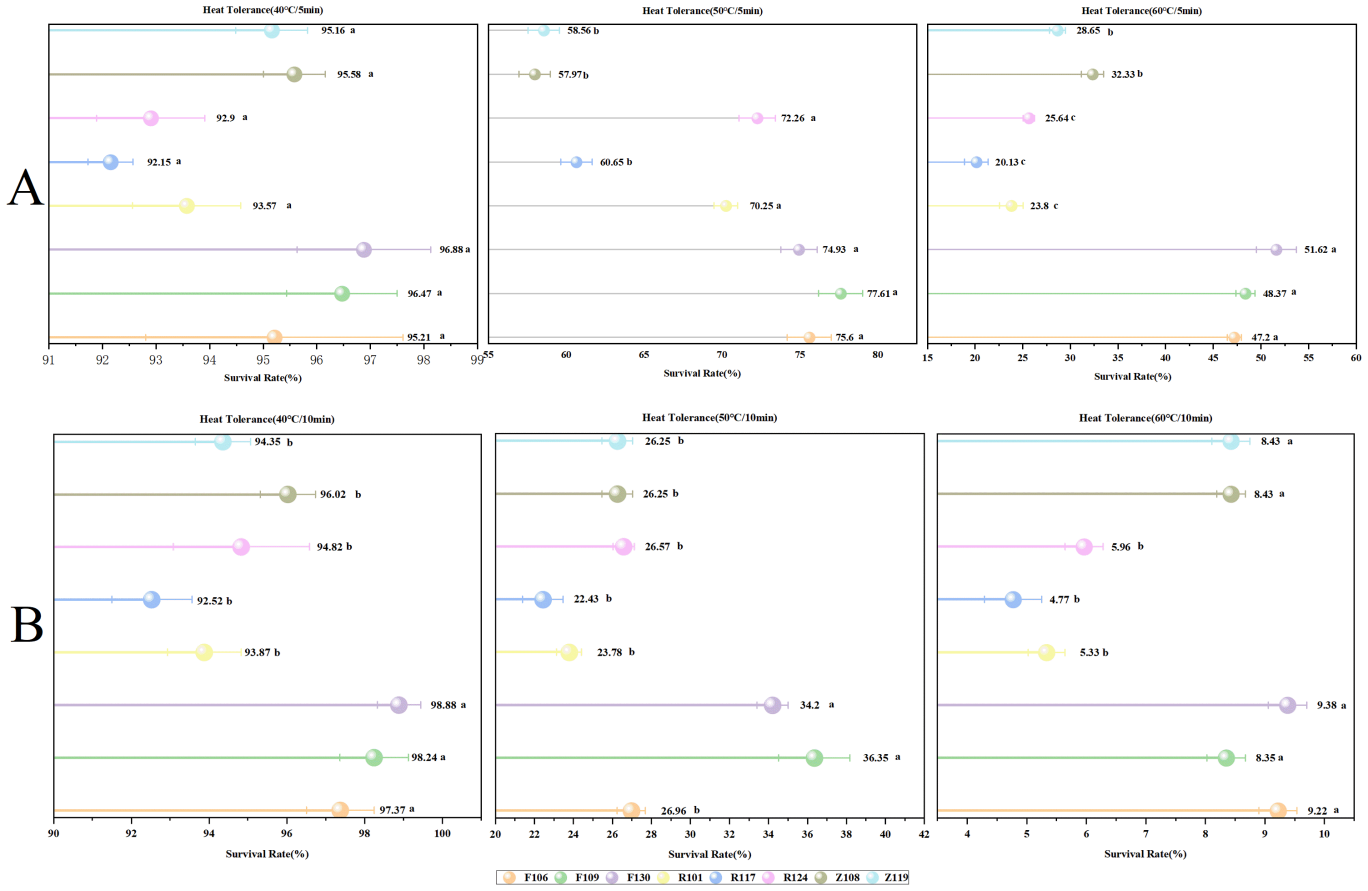
Assessment of the thermal tolerance of the isolated lactic acid bacteria strains. Strains were treated at different temperatures (40 °C–60 °C) for (A) 5 min and (B) 10 min. The different lowercase letters (*e.g.*, a, b, c, d) presented in the figures indicate statistically significant differences among the eight isolates within the same experimental group at the *p* < 0.05 level. Differences were considered statistically significant when the *p*-value was less than 0.05.

The results of stress tolerance tests showed that *Pediococcus* strains (R101, R117) and *Lactobacillus* strains (Z108, Z119) exhibited stronger tolerance to acidic environments compared to *Enterococcus* strains (F106, F109, F130) (Fig. 3A). The survival rate of *Enterococcus* strains was only 14% ± 0.13% at pH 2.0. In bile salt tolerance tests, *E. faecium* F130 displayed the highest viability (6.28%) at a 0.5% (w/v) bile salt concentration (Fig. 3B). Heat stress testing showed that F130 maintained 51.62% viability after 5 min at 60 °C, demonstrating a significant heat tolerance advantage. However, after 10 min at 60 °C, the survival rate of all strains dropped below 10% (Fig. 4).

#### Cell surface hydrophobicity and coaggregation and adhesion characteristics

**Figure 5:**
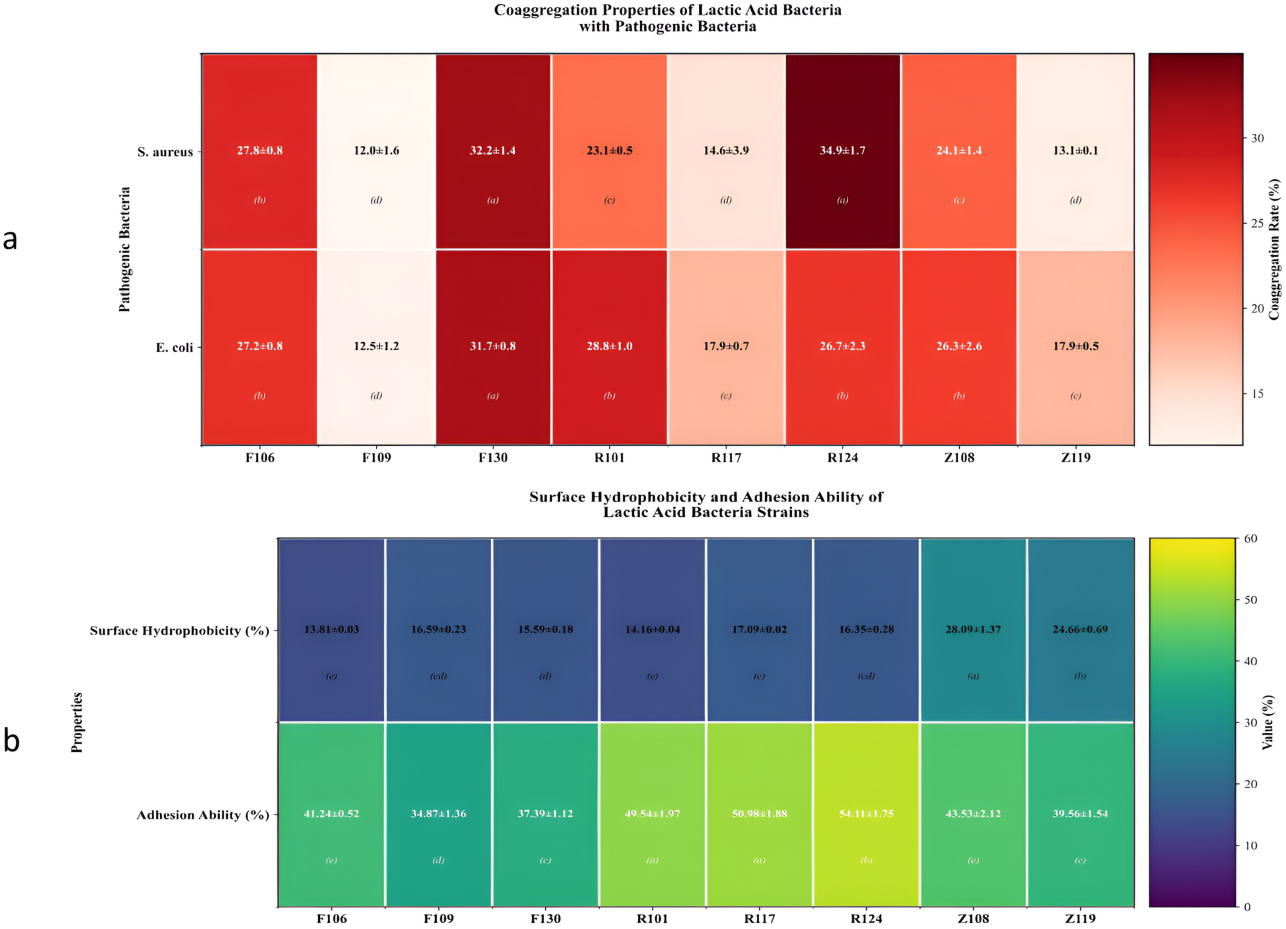
Co-aggregation and colonization characteristics. (A) Co-aggregation rate with *Staphylococcus aureus* (*S. aureus*) and *Escherichia coli* (*E. coli*). (B) Cell surface hydrophobicity and adhesion ability to IPEC-J2 cells. The different lowercase letters (*e.g.*, a, b, c, d) presented in the figures indicate statistically significant differences among the eight isolates within the same experimental group at the *p* < 0.05 level. Differences were considered statistically significant when the *p*-value was less than 0.05.

In terms of cell surface hydrophobicity, *L. plantarum* Z108 showed the best performance (28.09%), while *E. faecium* F106 had the lowest (13.81%). Coaggregation potential (Fig. 5) revealed strain-specific differences. *P. pentosaceus* R124 exhibited the highest coaggregation rate with *S. aureus* (34.9%), while *E. faecium* F130 showed the strongest coaggregation with *E. coli* (31.7%). *E. faecalis* F109 showed the weakest coaggregation in both tests (12% and 12.5%, respectively). In the IPEC-J2 cell adhesion assay, R124 showed the best adhesion (54.11%), followed by *P.acidilactici* R117 (50.98%). The adhesion rate of R124 was significantly higher than that of F109 (34.87%). These results highlight differences in intestinal colonization potential among the strains.

#### Biochemical identification

Biochemical analysis showed that all strains could hydrolyze esculin (Table 3). The strains belonging to *Enterococcus* (F106, F109, F130) and *Pediococcus* (R101, R117) exhibited the broadest metabolic capabilities, fermenting all nine tested carbohydrates. *P. pentosaceus* R124 had a similar metabolic profile but could not ferment cellobiose. *L. plantarum* strains (Z108, Z119) displayed metabolic diversity, with Z108 having the most restricted metabolic profile, unable to utilize melezitose, sorbitol, and mannitol.

### Whole genome analysis of representative strains

Based on all experimental results, *E. faecium* F130, *P. pentosaceus* R124, and *L. plantarum* Z108 were selected for whole-genome sequencing to represent the different safety risks and probiotic potentials observed in the isolated strains. F130 was chosen as the high-risk representative mainly due to its significant multidrug resistance phenotype and strong bile salt tolerance, which reflects its ability to adapt to the gut environment. R124 and Z108 were selected as promising probiotic candidates because they not only exhibit lower safety risks but also demonstrate excellent functional characteristics.

#### Genome overview and annotation

**Figure 6:**
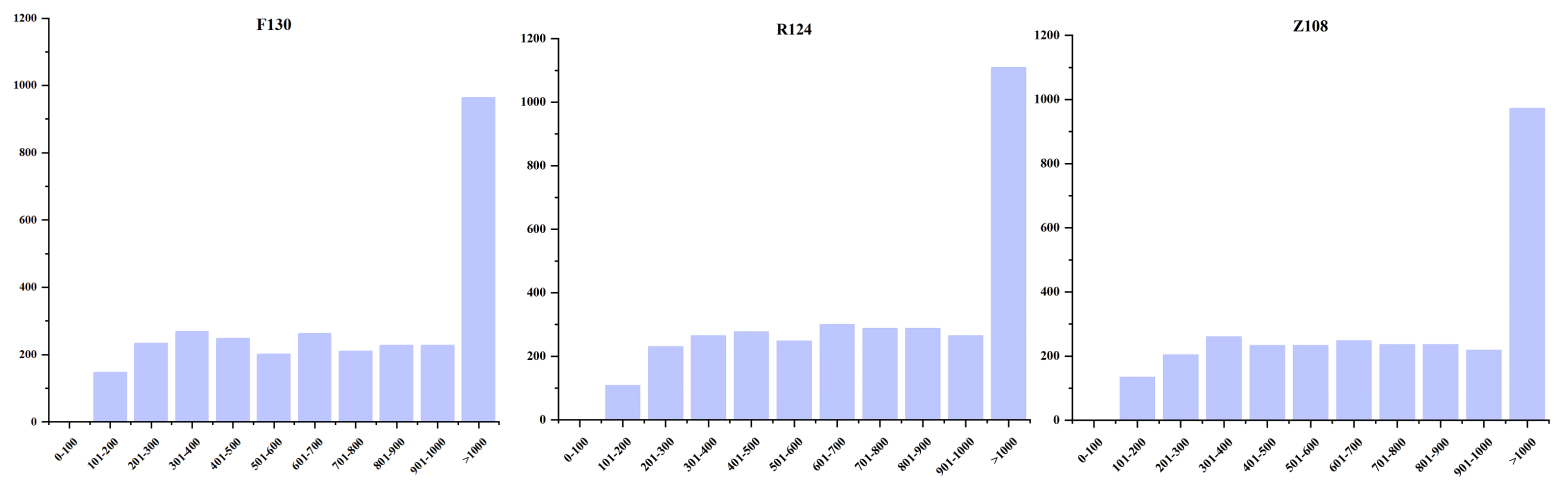
Length distribution of protein-coding genes in whole-genome sequenced strains.

The genome of *E. faecium* F130 is 3.09 Mb and consists of one chromosome and seven plasmids, with a GC content of 38.25%, and contains 3,015 predicted protein-coding sequences. The genome of *L. plantarum* Z108 is 3.21 Mb and consists of one chromosome and one plasmid, with a GC content of 44.57%, and contains 3,011 coding sequences. *P. pentosaceus* R124 has the simplest genome structure with a size of 1.73 Mb, consisting of a single chromosome, with no plasmids detected, a GC content of 37.24%, and 1,698 protein-coding sequences, which account for 88.13% of the total genome sequences. The length distribution of the protein-coding sequences in the three strains is shown in Fig. 6.

In terms of functional genome annotation, we focused on analyzing ARGs and VFGs, which are closely related to probiotic safety. No antibiotic resistance or VFGs were detected in the genomes of *P. pentosaceus* R124 and *L. plantarum* Z108. In contrast, the genome of *E. faecium* F130 contained 4 VFGs and 13 ARGs, with eight of these ARGs located on plasmids (PlasmidA and PlasmidE). A detailed annotation of the virulence and resistance genes is provided in Table 4.

#### Mobile genetic elements and plasmid characteristics

Analysis of the MGEs in the genomes of the three strains revealed that the genome of *P. pentosaceus* R124 is highly stable, containing only a small number of MGEs (two gene islands, one prophage, and three transposase sequences). *L. plantarum* Z108 contains a greater number of MGEs (eight gene islands, three prophages, and seven transposons, among others). In contrast, *E. faecium* F130′s genome carries a large number of MGEs, including 25 gene islands, four prophages, 51 transposons, and 37 insertion sequences. Detailed characteristics of these elements can be found in Tables S1 and S2.

**Table 3:** Biochemical identification results of the isolated lactic acid bacteria strains.

**Strain**	**Aesculin**	**Raffinose**	**Lactose**	**Sorbitol**	**Salicin**	**Mannitol**	**Maltose**	**Cellobiose**	**Sucrose**
**F106**	+	+	+	+	+	+	+	+	+
**F109**	+	+	+	+	+	+	+	+	+
**F130**	+	+	+	+	+	+	+	+	+
**R101**	+	+	+	+	+	+	+	+	+
**R117**	+	+	+	+	+	+	+	+	+
**R124**	+	+	+	+	+	+	+	–	+
**Z108**	+	–	+	–	+	–	+	+	+
**Z119**	+	–	+	+	+	+	+	+	+

**Notes.**

The concentration used for all substrates was 0.5%. “+” denotes a positive result; “−” denotes a negative result.

**Table 4:** Virulence factors and antibiotic resistance genes identified in *Enterococcus faecium* F130.

Type	Gene name	Location	Category/Mechanism
Virulence Factors	*bopD*	Chromosome	Biofilm
	*SgrA*	Chromosome	Adherence
	*Acm*	Chromosome	Adherence
	*Scm*	Chromosome	Adherence
Antibiotic Resistance Genes	*AAC(6′)-II*	Chromosome	Antibiotic inactivation
	*ANT(9)-Ia*	Chromosome	Antibiotic inactivation
	*efmA*	Chromosome	Antibiotic efflux
	*ErmT*	Chromosome	Antibiotic target alteration
	*tet(M)*	Chromosome	Antibiotic target protection
	*AAC6_Ie_APH2_Ia*	PlasmidA	Antibiotic inactivation
	*AAC6_Ie_APH2_Ia*	PlasmidA	Antibiotic inactivation
	*ANT(6)-Ia*	PlasmidA	Antibiotic inactivation
	*dfrG*	PlasmidA	Antibiotic target replacement
	*ErmB*	PlasmidA	Antibiotic target alteration
	*fexA*	PlasmidA	Antibiotic efflux
	*lsaE*	PlasmidA	Antibiotic target protection
	*ANT(4′)-Ib*	PlasmidE	Antibiotic inactivation

**Notes.**

The gene ARO:3002597 is present in two copies on PlasmidA.

Notably, among the seven plasmids in *E. faecium* F130, PlasmidE raised particular concern. This plasmid, annotated as being derived from *S. aureus*, contains aminoglycoside resistance genes and anti-toxin genes, indicating F130′s potential to spread resistance genes. *L. plantarum* Z108′s PlasmidH encodes a site-specific recombinase, PemI-like and PemK/MazF family toxins, and IS30/IS5 family transposases, highlighting its potential role in horizontal gene co-transfer and stress response. Detailed information is available in Table S3.

### Pan-genome analysis of representative strains

#### Pan-genome structure and features

A pan-genome comprising 280 *E. faecium*, 96 *P. pentosaceus*, and 127 *L. plantarum* strains was constructed by screening public databases and integrating the strains sequenced in this study (see Tables S4–S6 for strain IDs). As shown in Table 5, the pan-genomes of the three species were open. *E. faecium* exhibited the lowest proportion of core genes (4.82%) and the highest proportion of cloud genes (79.3%). *P. pentosaceus* had the lowest core gene proportion (1.91%), while *L. plantarum* had the relatively highest proportion of core genes (8.31%).

**Table 5:** Structural characteristics of the pan-genomes of *Enterococcus faecium* (*E. faecium*), *Pediococcus pentosaceus* (*P. pentosaceus*), and *Lactiplantibacillus plantarum* (*L. plantarum*).

Sample name	Core genes	Soft core genes	Shell genes	Cloud genes	Total
*E. faecium*	877	414	2477	14,442	18,210
*P. pentosaceus*	138	685	1319	5076	7218
*L. plantarum*	1216	434	2271	10,698	14,619

**Notes.**

Core genes: present in ≥ 99% of strains; Soft core: ≥ 95% to <99%; Shell: ≥ 15% to <95%; Cloud: <15%.

#### Annotation of antibiotic resistance genes and virulence factor genes in the pan-genome

The annotation of ARGs revealed significant inter-species differences in detection rates. Under strict annotation thresholds (Identity >95%, Coverage >95%, *E*-value ≤ 1e−5), all 280 *E. faecium* strains were found to harbor ARGs, with the number of genes ranging from 1 to 18. In contrast, only 6.2% (6/97) of *P. pentosaceus* strains were annotated with ARGs, involving eight different genotypes. *L. plantarum* showed the lowest detection rate for ARGs, with only 1.6% (2/127) of strains carrying the *tet(M)* gene. Further details can be found in Table S7.

The distribution of VFGs followed a similar pattern. Using a more lenient annotation threshold (Identity >85%, Coverage >85%, *E*-value ≤ 1e−5), 100% of *E. faecium* strains were annotated with VFGs, with the number of genes ranging from 6 to 23. In contrast, no *P. pentosaceus* strains were annotated with any known VFGs. VFGs were also rare in *L. plantarum*, with only 1.6% (2/127) of strains annotated with the *STER_RS05250* and *waaB* genes. Detailed information is available in Table S8.

#### Analysis of mobile genetic elements and plasmids in the pan-genome

The MobileOG database classifies MGEs into five categories: integration/excision (IE), phage (P), replication/recombination/repair (RRR), stability/transfer/defense (STD), and transfer (T). The analysis of MGEs in the pan-genome revealed that *E. faecium* strains generally carry a higher number of related genes compared to *P. pentosaceus* and *L. plantarum*, particularly in the functional categories related to T and IE (Table 6). Of particular note is the prevalence of plasmids. The *E. faecium* pan-genome identified 810 plasmid-related genes across 267 strains. In contrast, only 15 *P. pentosaceus* strains in the pan-genome carried 18 plasmid-related genes. The *L. plantarum* pan-genome identified 659 plasmid-related genes across 124 strains. Further details are provided in Tables S9–S14.

**Table 6:** Average number of mobile genetic element (MGE) genes per strain in the pan-genomes of *Enterococcus faecium* (*E. faecium*), *Pediococcus pentosaceus* (*P. pentosaceus*) and *Lactiplantibacillus plantarum* (*L. plantarum*).

Sample Name	RRR	P	T	STD	IE
*E. faecium*	68.88	43.1	30.3	21.48	79.53
*P. pentosaceus*	4.42	6.39	2.6	2.59	11.6
*L. plantarum*	10.3	23.77	6.95	5.13	29.04

**Notes.**

Values represent the mean number of genes per strain annotated to each MGE category.

RRR replication/recombination/repair P phage T transfer STD stability/transfer/defense IE integration/excision

### Experimental validation of gene level transfer

To experimentally validate the horizontal transfer of resistance genes, *E. faecium* F130 was used as the donor strain and *E. faecalis* JH2-2 as the recipient strain in a unidirectional conjugation experiment. The filter-mating method was employed, and the results showed that, under antibiotic selection pressure, plasmids were spontaneously transferred to the recipient strain, with a conjugation frequency of 1.03  ×  10^−^^6^. The conjugants acquired dual resistance to kanamycin and rifampicin, which is consistent with the resistance markers carried by PlasmidE. This phenomenon indicates that *E. faecium* F130 has the ability to mediate the horizontal spread of resistance factors and provides experimental evidence for the mobility of PlasmidE in these pig farm-derived strains within a specific context. The original gel images used for molecular validation can be found in Fig. S9.

## Discussion

LAB are crucial components of the animal gut microbiota and confer various beneficial properties (De Vos et al., 2022). Probiotics derived from the host often adapt more effectively to the host’s intestinal environment (Chen et al., 2023). To assess the safety and probiotic potential of pig-derived LAB, this study isolated strains from fecal samples collected from a large-scale pig farm in Anhui Province and conducted a systematic safety evaluation. Whole-genome sequencing revealed potential risks in pig-derived LAB, including antibiotic resistance, virulence gene carriage, and HGT. Additionally, strains with promising probiotic potential and high safety were identified.

The functionality of LAB depends on their growth and metabolic activities (Ahlberg et al., 2017). We observed that *Enterococcus* strains exhibited shorter growth cycles and more significant pH decreases, suggesting they may colonize the gut more rapidly and influence the intestinal environment (Rahayu et al., 2021). Genome analysis showed that *E. faecium* F130 appears to possess substantial carbohydrate metabolism capacity, providing energy and metabolic prerequisites for rapid growth and organic acid production. Probiotics must endure the dual challenges of strong stomach acidity (pH 2.0–3.0) and intestinal bile salts (0.03%–0.3%) (Tian et al., 2015; Wang et al., 2019). *In vitro* acid tolerance tests demonstrated that *P. pentosaceus* (up to 24.5%) and *L. plantarum* (up to 24.18%) exhibited significantly better tolerance under pH 2.0 conditions compared to *Enterococcus* strains (up to 14.02%), a result consistent with findings from Moussaid et al. (2023). The high acid tolerance of *P. pentosaceus* R124 and *L. plantarum* Z108 may be partly attributed to the complete F-type H+ ATPase system (K02111–K02115) and molecular chaperones such as groEL (K04077), which help maintain pH homeostasis and protein stability (Fourie & Wilson, 2020; Sasaki et al., 2014). These functional genes allow these strains to better cope with acidic environments and maintain intracellular pH balance, potentially enhancing their survival in the gastrointestinal tract.

Bile salt tolerance is another key criterion for screening high-quality probiotic strains (Liang et al., 2024). Bile salts can disrupt phospholipids and proteins in the cell membrane, impair membrane integrity and cellular homeostasis, ultimately leading to cell damage and death (Begley, Gahan & Hill, 2005). Despite the generally lower acid tolerance of *Enterococcus* strains, *E. faecium* F130 exhibited the highest survival rate (6.28%) under high-concentration bile salt conditions (5 g/L), simulating the harsh environment of the duodenum. This notable difference in tolerance suggests that F130 may possess specialized membrane protection mechanisms or bile salt hydrolase activity (Babu et al., 2004). Genome analysis supported this possibility. The F130 genome contains a *bsh* gene (K01442, bile salt hydrolase), which encodes an enzyme that hydrolyzes bile salts, reducing their damaging effects on the cell membrane (Begley, Hill & Gahan, 2006). Studies have shown that the *bsh* gene is commonly found in many LAB species and is closely related to their bile salt tolerance (Bustos et al., 2012; Lv et al., 2024). Additionally, the abundant ABC transporters (189 genes related to membrane transport) in F130 further promote the active efflux of bile acids, minimizing bile salt damage to the cell membrane, potentially enhancing the strain’s bile salt tolerance. ABC transporters are considered key mechanisms by which LAB respond to bile salt stress in the intestinal environment (Zhu et al., 2024).

As probiotics, candidate strains must be able to colonize the host’s intestine, which mainly depends on their ability to adhere to small intestine mucosa and epithelial cells (Ouwehand et al., 1999). This ability allows probiotics to successfully colonize the host gut by antagonizing the adhesion of pathogenic bacteria, thereby exerting their beneficial effects (Oh, Daliri & Oh, 2018). In this study, we assessed the colonization potential of these LAB by measuring their cell surface hydrophobicity, auto-aggregation ability, and co-aggregation capacity with pathogenic bacteria. The results showed that *P. pentosaceus* R124 and *E. faecium* F130 exhibited the strongest co-aggregation ability with *S. aureus* and *E. coli*, which is consistent with previous reports (Oussaief et al., 2020), indicating that co-aggregation capacity is strain-dependent and pathogen-dependent (Ben-Miled et al., 2023). Compared to strains of *Pediococcus* and *Enterococcus* tested by Osmanagaoglu, Kiran & Ataoglu (2010) and Reuben et al. (2019), the strains in this study demonstrated stronger co-aggregation with the pathogenic bacteria. R124 had the highest adhesion rate to IPEC-J2 cells (54.11%). This result suggests that R124 can effectively prevent pathogen attachment through competitive exclusion mechanisms, a fundamental feature for probiotic colonization (Du et al., 2022; Knipe et al., 2021). The excellent adhesion ability of R124 may be closely related to its high expression of cell surface proteins or hydrophobic structures (Adnan et al., 2021; Xue et al., 2021), indicating it may be a candidate with considerable colonization potential (Sadeghi et al., 2022). Although the adhesion rate of *L. plantarum* Z108 was slightly lower than that of R124, its cell surface hydrophobicity was the highest among all strains (28.09%). Combined with its excellent acid tolerance, Z108 may serve as another functionally complementary probiotic candidate (Fentie et al., 2024).

Safety assessment of LAB is a critical step in determining their probiotic potential. Hemolytic activity is commonly associated with hemolysins produced by pathogenic bacteria, which can disrupt the host’s red blood cell membranes (Di Vincenzo et al., 2024). In this study, all eight LAB strains exhibited a γ-hemolytic phenotype, which aligns with the basic safety assessment requirements set by FAO/WHO for probiotics. Numerous studies have indicated that probiotics are generally expected not to exhibit hemolytic activity (Pon Additives et al., 2018; Stefańska et al., 2021).

In modern large-scale animal farming, LAB are often exposed to long-term antibiotic selection pressures. Pig-derived LAB, being subjected to strong antibiotic selection pressure, may potentially act as intermediaries in the transmission of resistance within the “One Health” framework (Jezak & Kozajda, 2022). Large-scale pig farming serves as a key source for the generation and spread of antibiotic-resistant genes (ARGs), contributing to a diverse gene pool (Chen et al., 2025). Compared to LAB from natural environments, pig-derived LAB are typically exposed to stronger antibiotic selection pressures, which may result in distinctive characteristics regarding antibiotic resistance (George et al., 2018). LAB from natural environments may exhibit lower frequencies of antibiotic resistance and virulence factor carriage because their growth environments are generally not subjected to the same intense antibiotic selection pressures (Wei, Zhou & Zhu, 2022). Our study found that pig-derived LAB strains frequently exhibited multi-drug resistance, especially with a high resistance rate to aminoglycoside antibiotics. This finding is in close agreement with the research by Duche et al. (2023), which noted that LAB strains commonly exhibit significant resistance to cephalosporins, aminoglycosides, quinolones, and glycopeptides (Zhang et al., 2023a; Zhang et al., 2023b). Resistance to aminoglycoside antibiotics is often considered intrinsic, determined by two factors: (1) the low permeability of the bacterial cell surface to aminoglycosides (Tian et al., 2025), and (2) the lack of cytochrome-mediated electron transport elements in LAB strains (Dušková et al., 2021). Notably, although *L. plantarum* Z108 and *P. pentosaceus* R124 exhibited a multidrug-resistant phenotype, no known acquired ARGs were detected in their genomes. Such inconsistencies between phenotype and genotype are not uncommon in lactic acid bacteria and are largely attributed to intrinsic resistance mechanisms rather than ARGs acquired *via* horizontal gene transfer. Because intrinsic resistance is generally not associated with horizontal gene transfer, it is usually considered low-risk in probiotic safety assessments. This finding underscores the importance of not only considering the probiotic characteristics of candidate strains but also thoroughly assessing their resistance profiles to ensure the safety of their long-term application.

Whole-genome sequencing analysis confirmed the high-risk features of *E. faecium* F130. The genome of F130 harbors four VFGs and 13 ARGs. The high density of resistance genes, particularly those located on plasmids, raises significant concerns, especially in pig farm environments. Studies have shown that, in such environments, resistance genes associated with MGEs tend to have higher expression efficiency and greater horizontal transmission potential compared to chromosomal loci (Zhang et al., 2023a; Zhang et al., 2023b). Recent global risk assessments have also indicated that resistance genes in animal manure share a high degree of genomic similarity with those found in human pathogens (Li et al., 2025). This suggests that the ARGs carried by F130 are not merely passive genomic features, but represent functional resistance determinants capable of transferring to human pathogens and increasing the risk of antimicrobial resistance in humans.

The four VFGs carried by F130 include three adhesion-related genes (*sgrA*, *acm*, *scm*) and one biofilm-related gene (*bopD*). The presence of these adhesion factors enhances F130′s colonization ability, especially in the gut environment. However, in *Enterococcus* species, these genes are also considered key factors in the transition from commensal to opportunistic pathogenic bacteria (Şchiopu et al., 2023). The expression level of the *acm* gene in *Enterococcus* is closely related to its pathogenicity (Nallapareddy et al., 2007), particularly when the bacteria enter the host’s immune-compromised environment. For example, the *scm* gene is an adhesion factor strongly associated with collagen binding (Sillanpää et al., 2008), and it has been validated in clinically isolated *Enterococcus* strains. *Scm* has a strong affinity for type V collagen, which is a key component of the intestinal submucosa (Rasheed et al., 2021). Therefore, the presence of *acm* and *scm* genes in F130 may influence its adhesion phenotype in various tissue environments, potentially affecting its interaction with the host and risk under specific conditions. Furthermore, the *bopD* gene in F130 is associated with biofilm formation. Biofilm formation allows bacteria to establish protective barriers against the host’s immune system, resist antibiotic treatment, and enhance their pathogenicity (Li et al., 2023; MacNair, Rutherford & Tan, 2024). While the *bopD* gene in F130 is not the same as the biofilm-forming mechanism mediated by *AtlAEfm* (a major autolysin in *E. faecium* that facilitates extracellular DNA release and surface protein anchoring), it reveals the synergistic relationship between adhesion factors and biofilm formation. The colonization mediated by *acm* and *scm* creates the conditions for biofilm formation driven by *bopD*, further enhancing F130′s colonization ability and resistance. Thus, although F130 may be introduced as a probiotic, its multiple adhesion and biofilm genes make it highly pathogenic in immunocompromised hosts or in adverse environments. More importantly, the presence of these genes suggests that F130 could potentially serve as a source of antibiotic resistance transmission under certain conditions, which may have implications for public health. In conclusion, while the virulence factors and resistance genes carried by F130 enhance its probiotic potential, they also expose it to the dual risk of being a potential pathogen and a source of antibiotic resistance transmission.

Notably, genome analysis revealed that more than half of the ARGs in *E. faecium* F130 are located on plasmids (Plasmid A and Plasmid E). Plasmids, as typical MGEs play a critical role in the horizontal transfer of antibiotic resistance within the gut microbiota (Zhang et al., 2022). Compared to chromosomally encoded genes, plasmid-borne ARGs exhibit significantly enhanced mobility, increasing their potential for horizontal dissemination (Partridge et al., 2018). Our filter-mating experiments further confirmed this potential, demonstrating that plasmid-borne ARGs could be successfully transferred to *E. faecalis* JH2-2, supporting the notion that F130 can act as a “genetic reservoir” in the pig gut, facilitating the spread of multidrug resistance through conjugation. This horizontal transfer mechanism not only elevates resistance in commensal bacteria but also poses a risk of transferring resistance genes to potential pathogens, thereby exacerbating the spread of antibiotic resistance.

To further evaluate the genetic characteristics of the selected strains, we conducted pan-genome analyses comparing them with conspecific strains from public databases. The pan-genome of *E. faecium* exhibited a low proportion of core genes (4.82%) and showed considerable variability in ARG content among strains (ranging from 1 to 18 genes). This genomic diversity indicates an open pan-genome, allowing for extensive gene gain and loss (Zhong et al., 2019). Compared to the species-wide average, F130 carries 13 ARGs, highlighting that its high resistance is not a general feature of *E. faecium* but rather a result of specific gene acquisitions, likely driven by antibiotic selection pressure in the farming environment (He et al., 2018). Moreover, the distribution of MGEs provides molecular evidence supporting the horizontal transfer observed *in vitro*, as the high density of integration- and transfer-related genes aligns with its role as a carrier of resistance genes.

Recent studies have increasingly emphasized the role of pig-derived LAB in resistance dissemination, particularly through MGE-mediated horizontal transfer of ARGs within the gut microbiota, which may contribute to the potential spread of resistance (Yang et al., 2020). These findings are consistent with recent assessments of antibiotic resistance risks in pig-derived LAB (Wang et al., 2023). In contrast, the genomes of *P. pentosaceus* and *L. plantarum* exhibit lower plasticity, and the detection rate of ARGs is very low, indicating that these species may exhibit comparatively higher genetic safety for probiotic development. Their low genomic variability and scarcity of resistance genes make them relatively safe with respect to gene transfer and resistance dissemination, supporting their potential as probiotics, particularly in environments with minimal antibiotic pressure.

Furthermore, the findings of this study offer important insights into the management of microbiomes in large-scale industrial production. Although this research initially focused on the screening and identification of specific isolates, the prevalence of multidrug resistance among the strains underscores the necessity of establishing systematic microbiome monitoring protocols within large-scale production pipelines for probiotics, food, or feed. To combat the spread of ARGs within production environments and end-products, future industrial practices should consider developing integrated approaches to control microbial communities, ensuring the biosafety and sustainability of large-scale bioproduction.

Although this study successfully reveals the safety spectrum of pig-derived LAB and experimentally validates the horizontal transfer of resistance genes, certain limitations must be acknowledged. First, the scope of the whole-genome sequencing analysis was limited, focusing only on three representative strains. This restricts our ability to fully capture the genetic diversity of all eight isolated strains, which in turn affects the generalizability of the genomic findings. Second, the study primarily focused on safety assessment, without systematically screening for key probiotic functional genes, such as those responsible for bacteriocin production or immune modulation. Consequently, our evaluation of the application potential of these candidate strains remains incomplete. Finally, the assessment of horizontal gene co-transfer and probiotic efficacy relied on *in vitro* experimental models. While these controlled studies provide strong mechanistic evidence, they cannot fully replicate the complex microbiota-host interactions in the gastrointestinal tract of live animals. Moreover, While the interactions between the isolates and pathogens were evaluated *via* coaggregation assays, direct *in vitro* antibacterial tests (*e.g.*, agar well diffusion) were not conducted to quantify the inhibition of pathogenic growth by secreted metabolites like bacteriocins. Therefore, while our results demonstrate the potential for competitive exclusion through physical interaction, the direct antimicrobial efficacy of these candidate strains requires further validation in future studies to fully characterize their probiotic potential. These limitations highlight the necessity of future research. Subsequent work should: (1) expand the scope of whole-genome sequencing to include more strains for a better characterization of genetic diversity; (2) include comprehensive screening for beneficial genes; (3) conduct *in vivo* studies to verify the occurrence of HGT and the functional properties of probiotics within the gut. Additionally, we will focus on studying pathogen inhibition and its molecular mechanisms as one of the next key research priorities. Addressing these gaps will enhance the translational value of this study’s conclusions in the development of safe probiotics.

## Conclusion

This study systematically evaluated the probiotic potential and biosafety of eight LAB strains isolated from a large-scale pig farm through *in vitro* functional assessments, whole-genome sequencing, and pan-genome analysis. Previous research has widely demonstrated the positive effects of LAB as alternatives to antibiotics in promoting animal health. However, the risk of LAB acting as potential reservoirs of ARGs has garnered increasing attention in the field of public health. The results of this study show that all eight isolated strains exhibited potential probiotic characteristics. Among them, *P. pentosaceus* R124 and *L. plantarum* Z108 displayed excellent adhesion abilities and acid tolerance *in vitro*, and notably lacked detectable ARGs and VFGs, indicating relatively high genetic safety and suggesting they may be promising probiotic candidates. At the same time, the study suggests potential safety concerns for LAB in specific ecological niches. The *E. faecium* F130 strain carried virulence genes, ARGs, and mobile resistance plasmids, which showed clear genetic divergence from its species and the other two genera. These findings may indicate the potential for horizontal transfer of multidrug resistance under certain conditions, suggesting a possible role as a “genetic reservoir,” but further *in vivo* studies are needed to confirm its actual impact. While this study provides a systematic reference for probiotic screening, some limitations remain. Future research will expand the scope of genome analysis and conduct *in vivo* experiments to verify the actual occurrence of probiotic efficacy and resistance gene transfer.

In conclusion, this study emphasizes the need to combine phenotypic function evaluation with comprehensive genomic safety assessments (particularly monitoring MGEs) during probiotic screening to ensure the long-term safety of their application.

### Availability of data and materials

Whole-genome sequence data for *Enterococcus faecium* F130, *Pediococcus pentosaceus* R124, and *Lactiplantibacillus plantarum* Z108 are publicly available in NCBI GenBank under BioProject PRJNA1253582. Genome assembly accessions are CP189633–CP189640 (F130_01), CP188822–CP188823 (Z108_01), and CP188821 (R124_01).

##  Supplemental Information

10.7717/peerj.21496/supp-1 Supplemental Information 1 Supplemental figures and tables

10.7717/peerj.21496/supp-2 Supplemental Information 2 Partial 16S rRNA gene sequences (from all strains)

10.7717/peerj.21496/supp-3 Supplemental Information 3 Raw data

10.7717/peerj.21496/supp-4 Supplemental Information 4 Type strain

10.7717/peerj.21496/supp-5 Supplemental Information 5 Distribution of antibiotic resistance genes

10.7717/peerj.21496/supp-6 Supplemental Information 6 Distribution of virulence genes

## Abbreviations

LAB: Lactic acid bacteria
K-B: Kirby-Bauer
ARGs: Antibiotic resistance genes
VFGs: Virulence factor genes
HGT: Horizontal gene transfer
MGEs: Mobile genetic elements

## Additional Information and Declarations

### Data Availability

The following information was supplied regarding data availability: The data is available in the Supplemental Files. The data is available at Zenodo:Li, S. (2025). In Vitro Probiotic Characteristics and Whole-Genome Sequencing Analysis of Porcine-Derived Lactic Acid Bacteria [Data set]. Zenodo. https://doi.org/10.5281/zenodo.17830260.
