# Supplementary material for: In vitro probiotic characteristics and whole-genome sequencing analysis of porcine-derived lactic acid bacteria
Source: PeerJ. 2026 Jul 15;14:e21496. doi: 10.7717/peerj.21496 (PMC13380237; doi:10.7717/peerj.21496)
Supplement: Supplemental Information 1 [file peerj-14-21496-s001.docx]

Supplementary Material

# Supplementary Figures and Tables

## Supplementary Figures
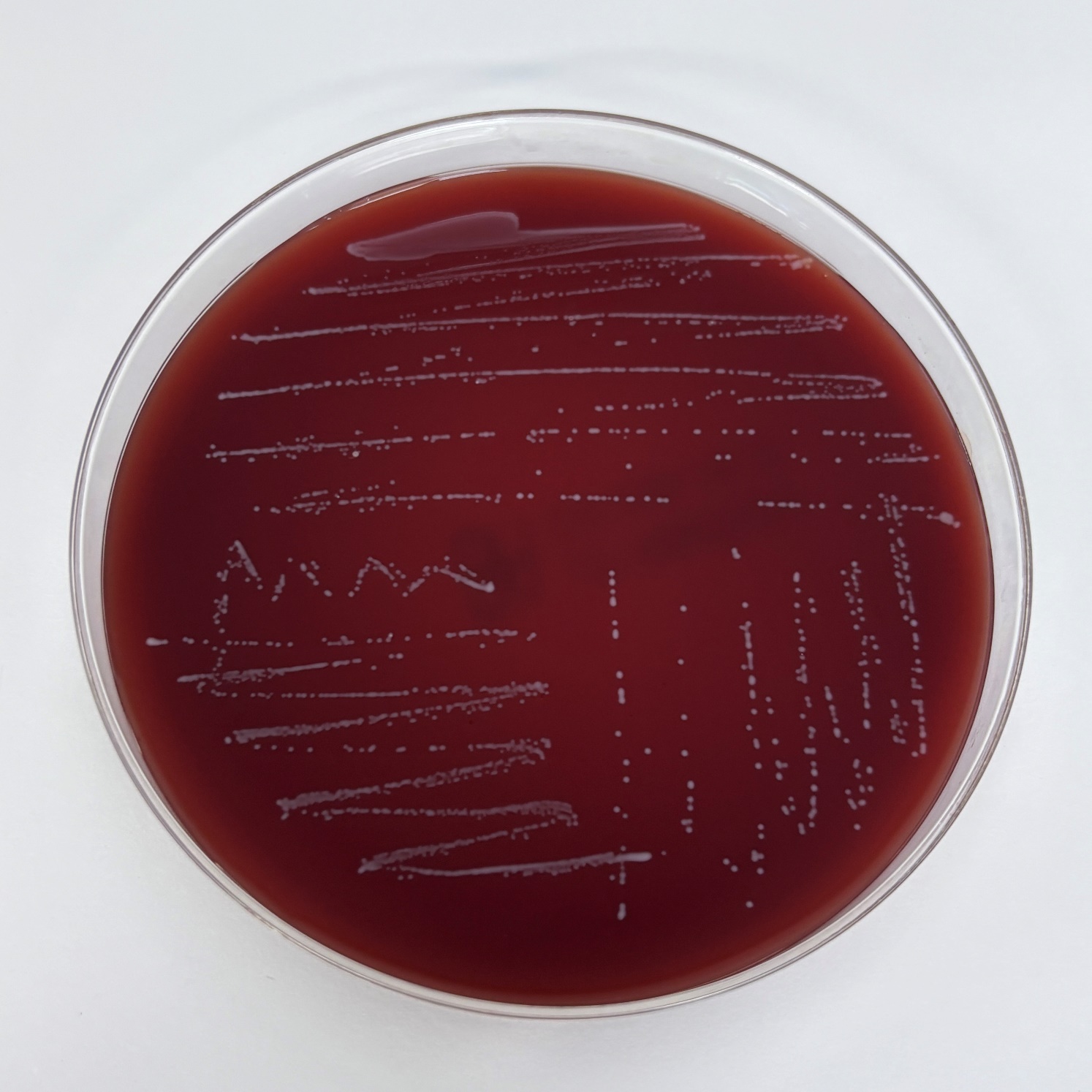
Supplementary Figure S1. Hemolytic activity of *Enterococcus faecium* F106 on blood agar plates.


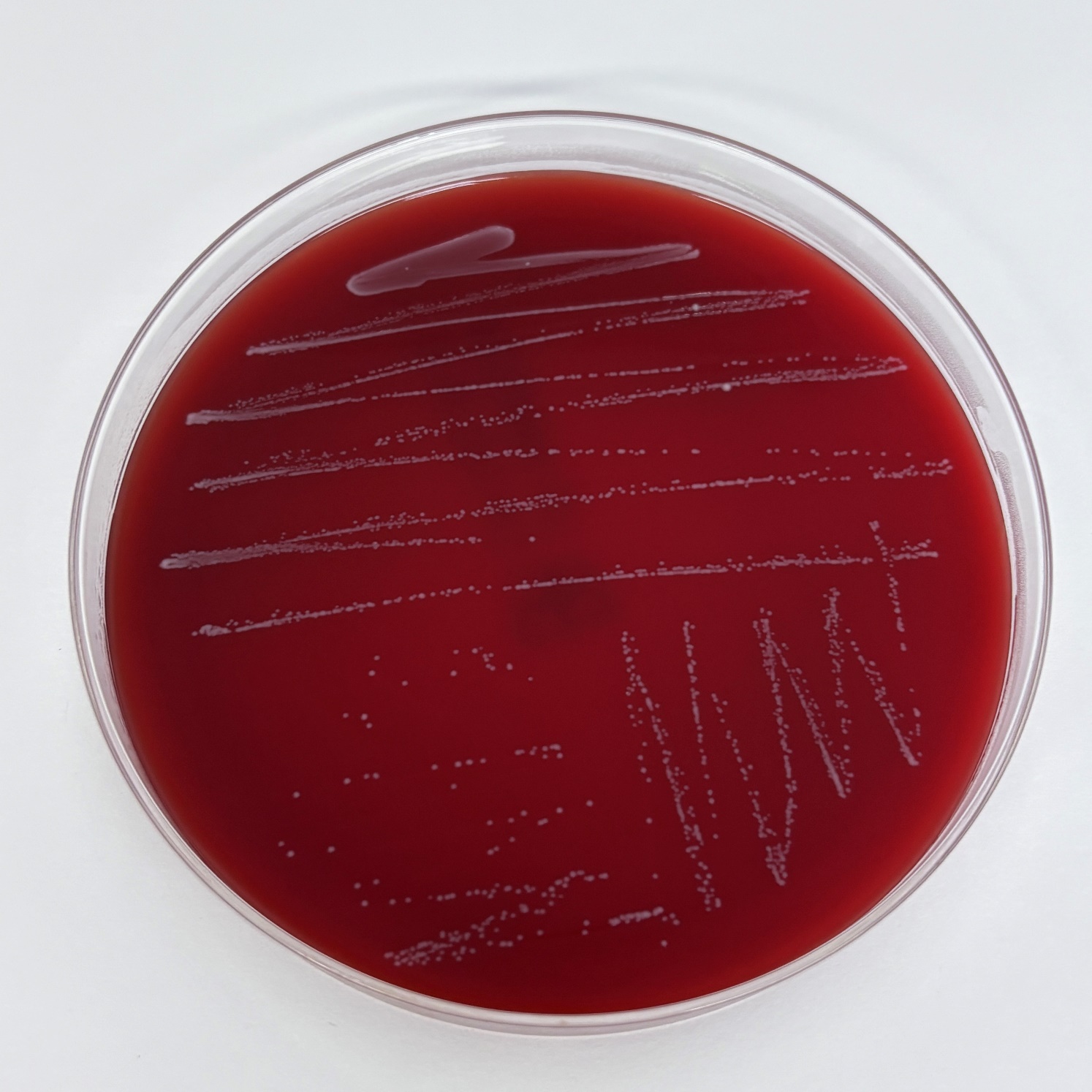


**Supplementary Figure S2.** Hemolytic activity of *Enterococcus faecalis* F109 on blood agar plates.


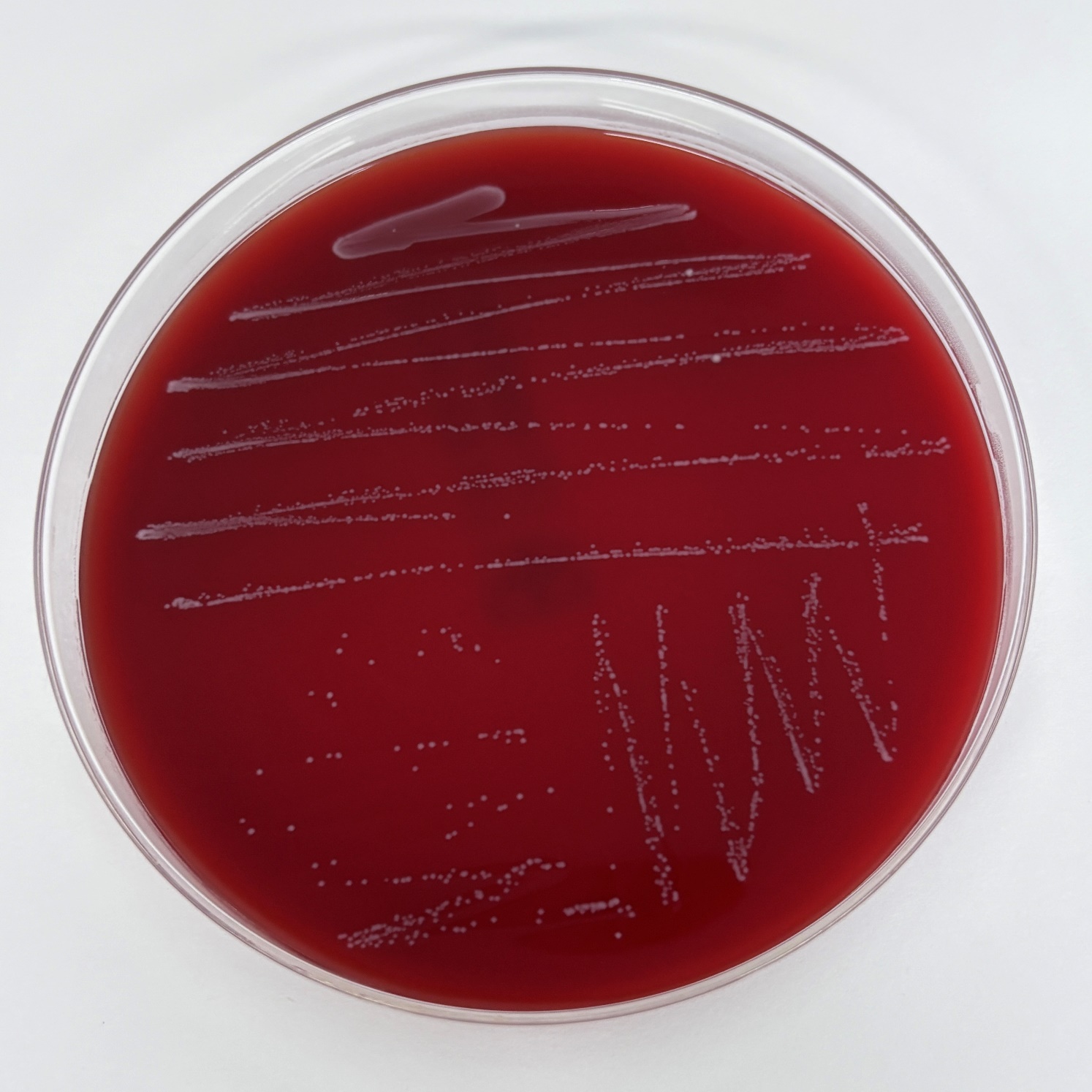


**Supplementary Figure S3.** Hemolytic activity of *Enterococcus faecium* F130 on blood agar plates.


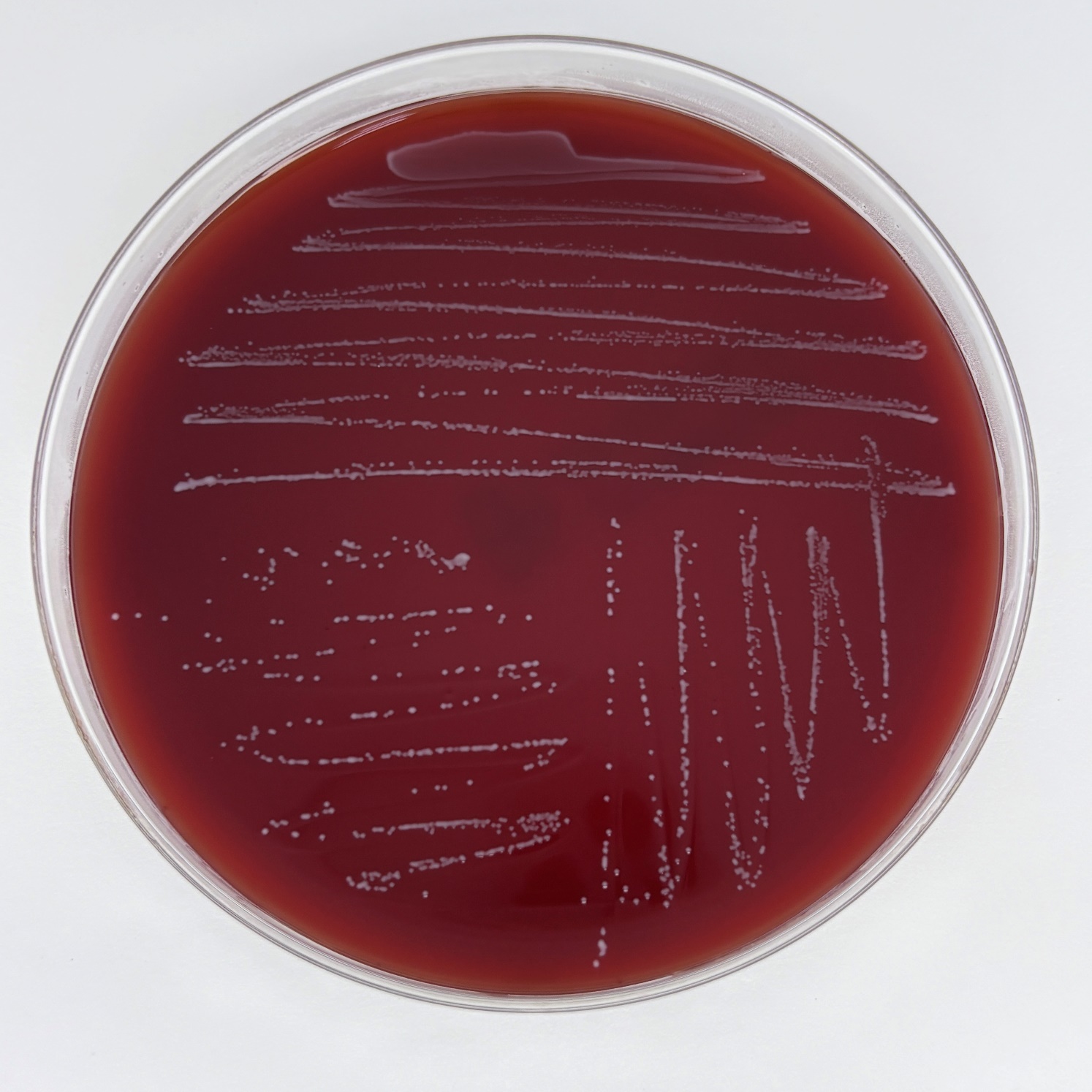


**Supplementary Figure S4.** Hemolytic activity of *Pediococcus acidilactici* R101 on blood agar plates.


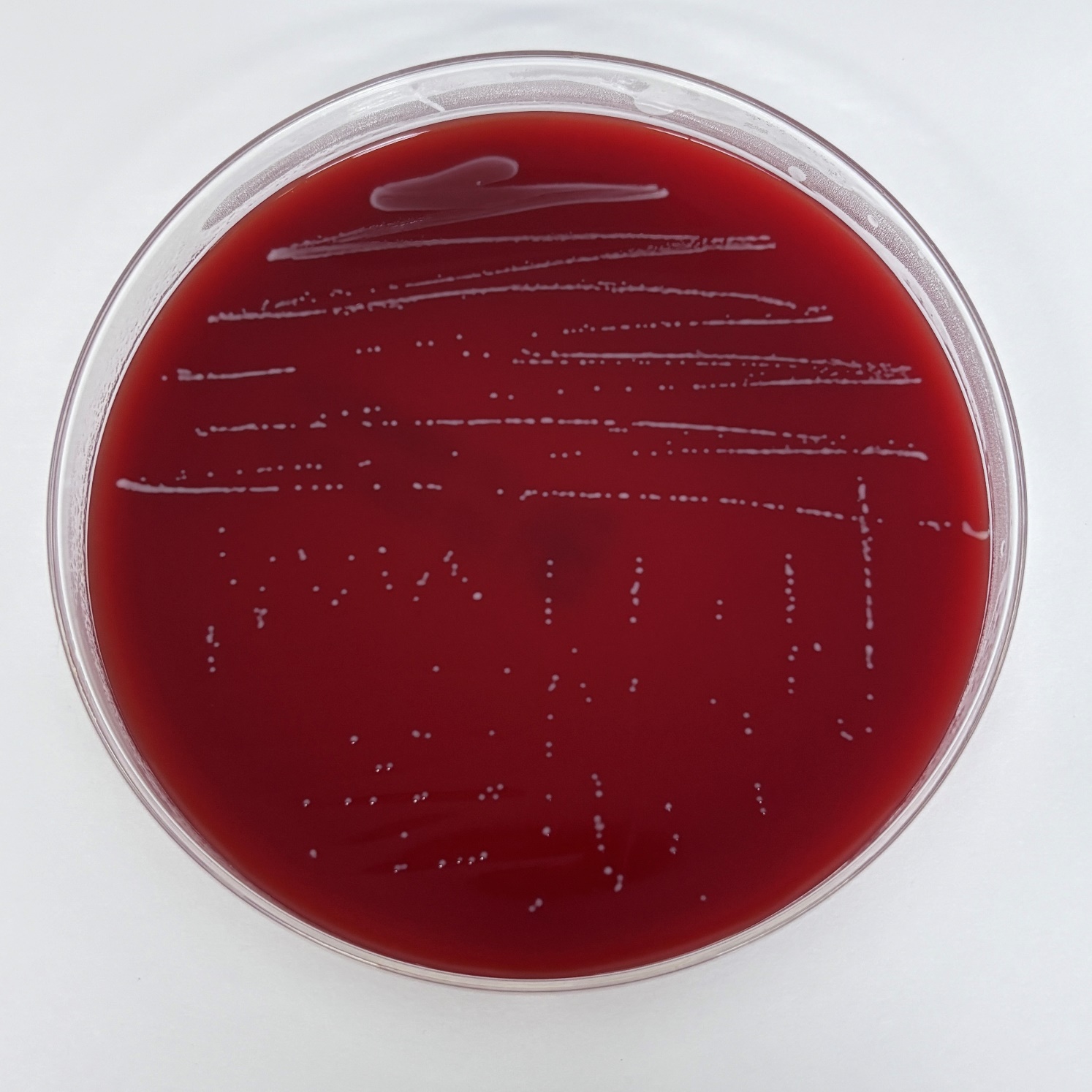


**Supplementary Figure S5.** Hemolytic activity of *Pediococcus acidilactici* R117 on blood agar plates.


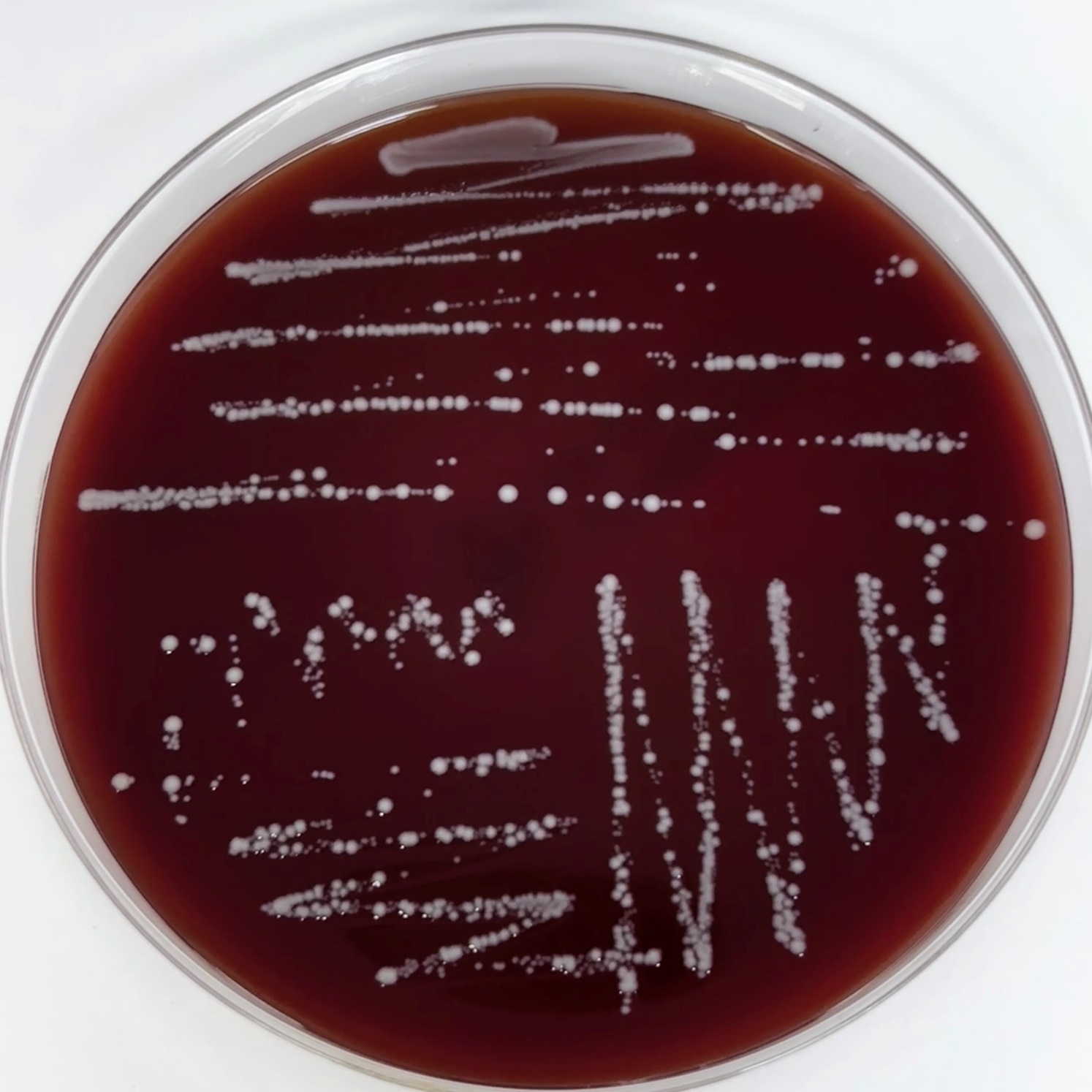


**Supplementary Figure S6.** Hemolytic activity of *Pediococcus pentosaceus* R124 on blood agar plates.


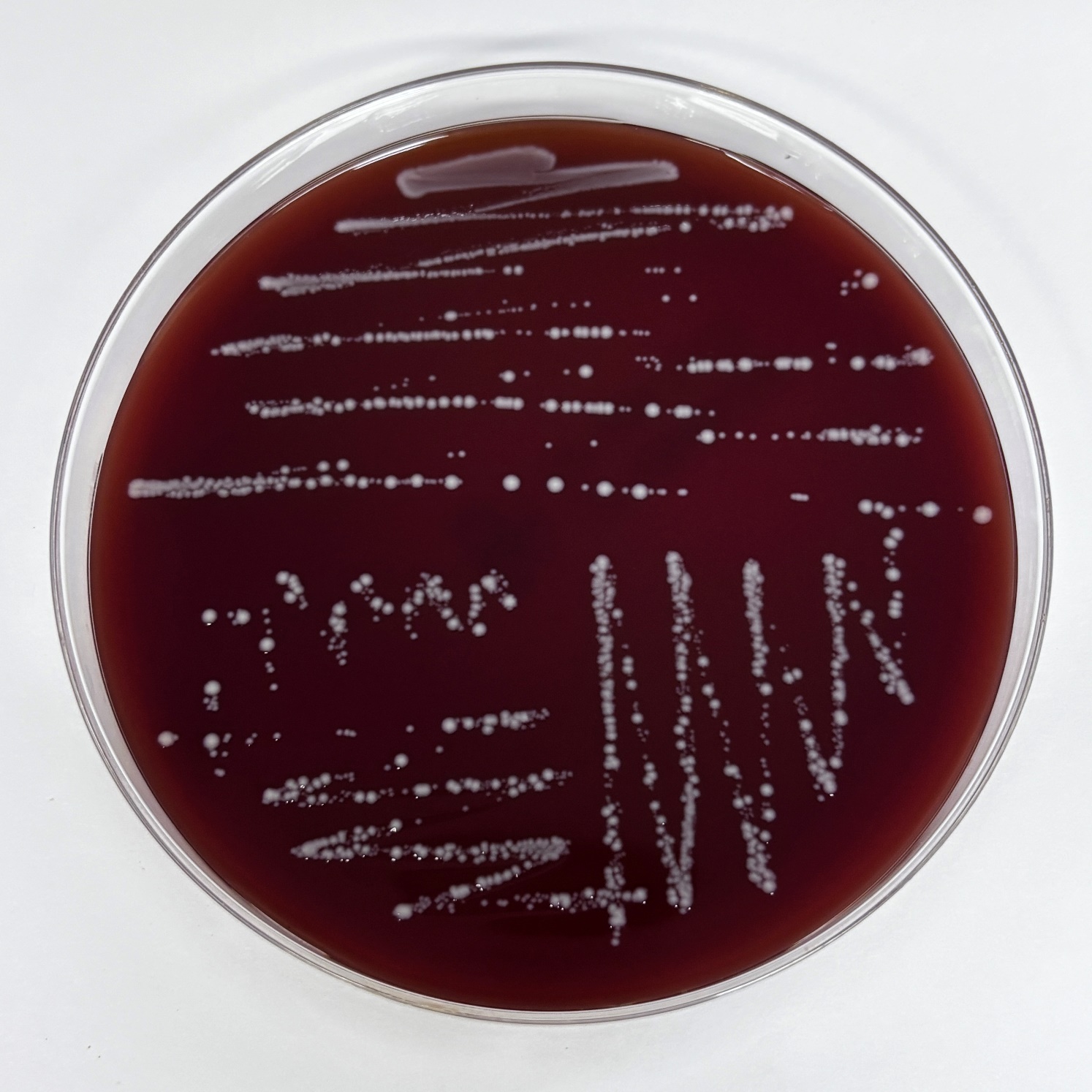


**Supplementary Figure S7.** Hemolytic activity of *Lactiplantibacillus plantarum* Z108 on blood agar plates.


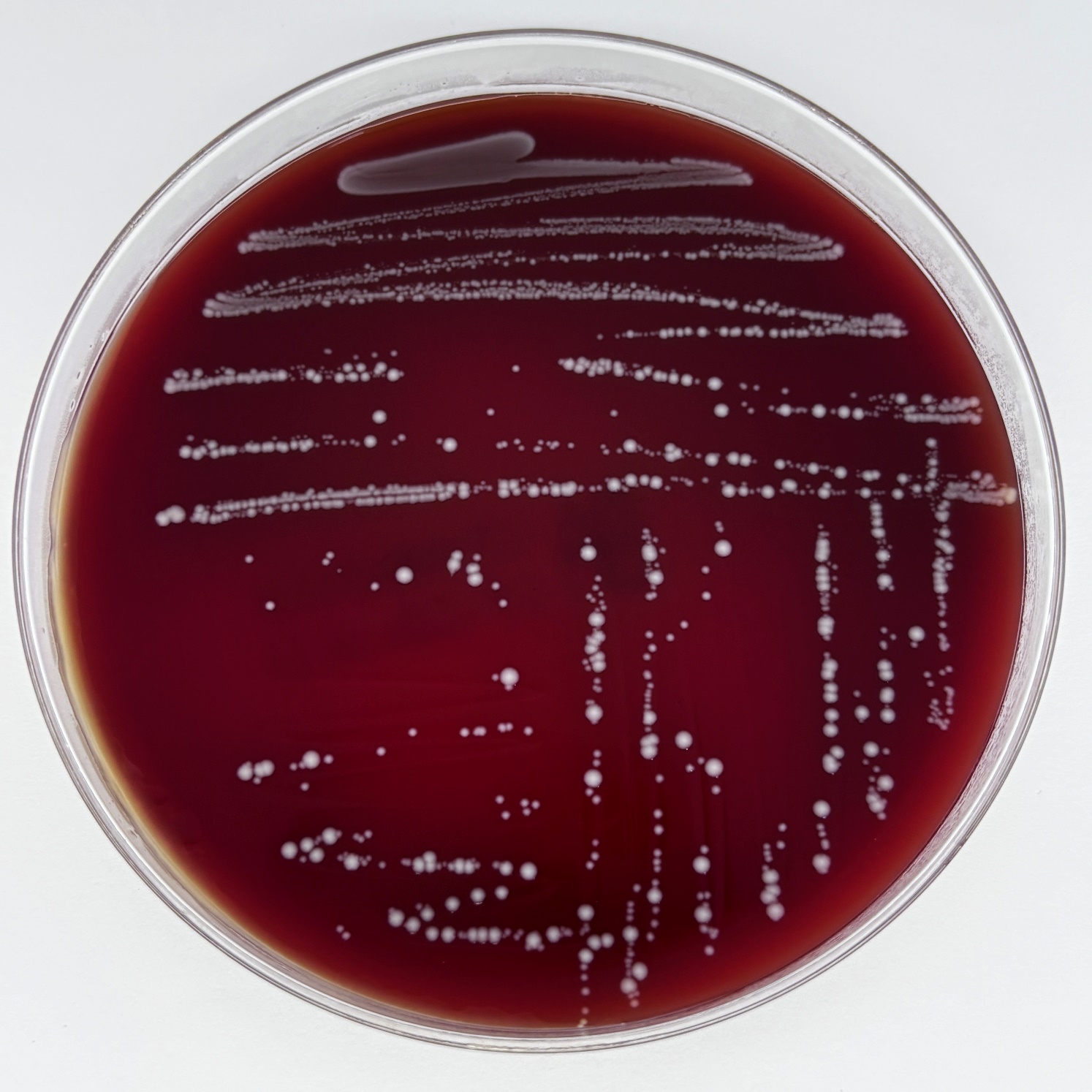


**Supplementary Figure S8.** Hemolytic activity of *Lactiplantibacillus plantarum* Z119 on blood agar plates.

**
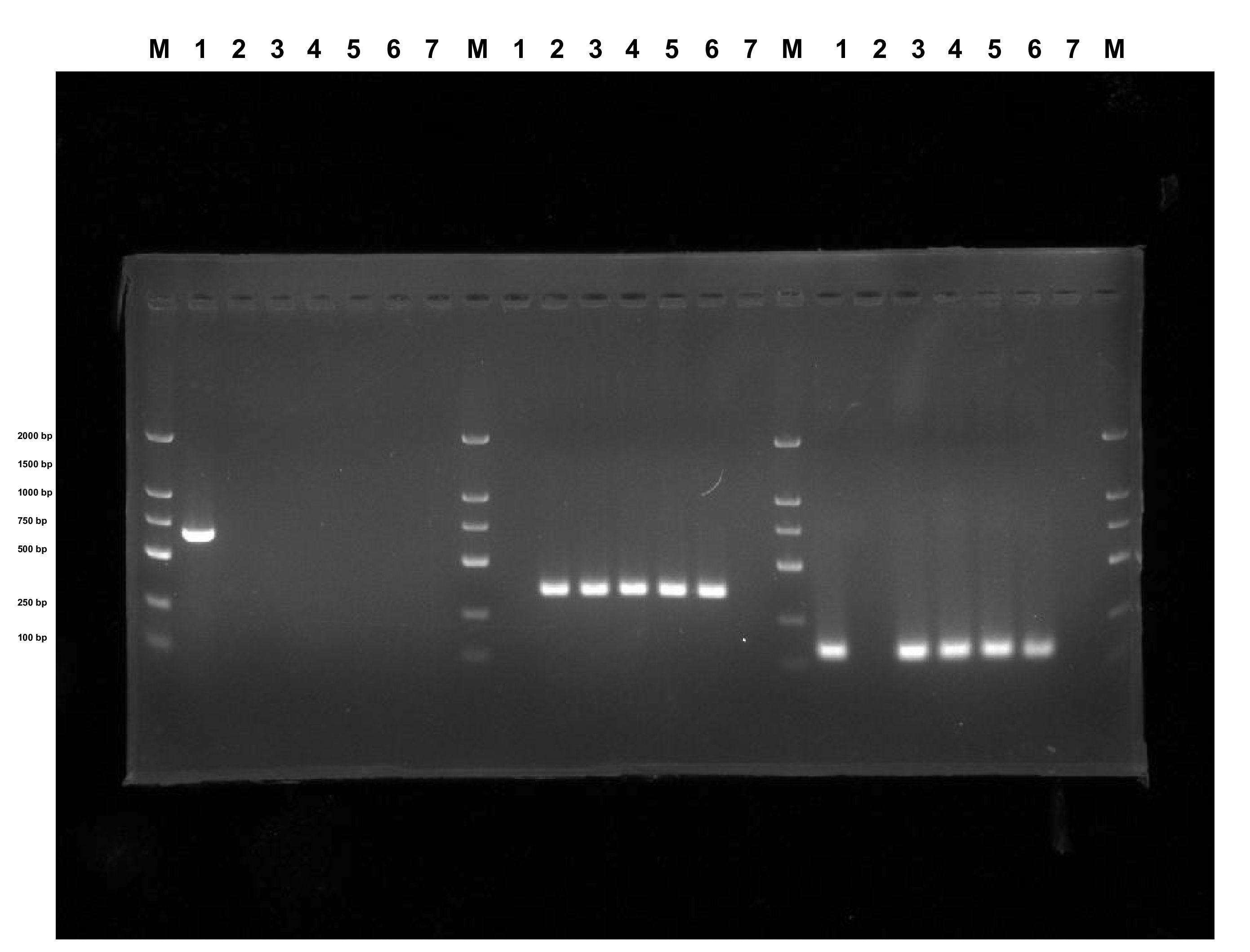
**

**Supplementary Figure S9.** Sealing Experiment Results. M: DNA Marker; 1: Donor *Enterococcus faecium* F130 (acting as the positive control); 2: Recipient strain JH2-2; 3-6: independent transconjugant colonies; 7: Negative control.

## Supplementary Tables

**Supplementary Table S1. Pre-phage&CRISPR predictions results.**

| Feature Type | Feature ID | Sample Name | Location | Start | End | Length (bp) | Additional Info |
| --- | --- | --- | --- | --- | --- | --- | --- |
| Prophage | Ph01 | F130 | Chromosome | 1012051 | 1044263 | 32213 | CDS No.: 52 |
|  | Ph02 | F130 | Chromosome | 1304143 | 1326711 | 22569 | CDS No.: 16 |
|  | Ph03 | F130 | Chromosome | 2018772 | 2051090 | 32319 | CDS No.: 47 |
|  | Ph04 | F130 | Chromosome | 2365199 | 2399255 | 34057 | CDS No.: 54 |
|  | Ph01 | R124 | Chromosome | 787571 | 823628 | 36058 | CDS No.: 48 |
|  | Ph01 | Z108 | Chromosome | 1173346 | 1209006 | 35661 | CDS No.: 49 |
|  | Ph02 | Z108 | Chromosome | 1810800 | 1837179 | 26380 | CDS No.: 33 |
|  | Ph03 | Z108 | Chromosome | 2142937 | 2176393 | 33457 | CDS No.: 49 |
| CRISPR | CRISPR1 | F130 | Chromosome | 1380059 | 1380279 | 221 | DR No.: 3, DR Len: 32, SPA Len: 62 |
|  | CRISPR2 | F130 | Chromosome | 2648917 | 2649149 | 233 | DR No.: 4, DR Len: 29, SPA Len: 39 |
|  | CRISPR3 | F130 | Chromosome | 2779778 | 2780516 | 739 | DR No.: 13, DR Len: 25, SPA Len: 34 |
|  | CRISPR1 | R124 | Chromosome | 57328 | 58030 | 703 | DR No.: 10, DR Len: 46, SPA Len: 27 |
|  | CRISPR1 | Z108 | Chromosome | 2719564 | 2719649 | 86 | DR No.: 2, DR Len: 23, SPA Len: 40 |

**Supplementary Table S2. Statistics of transposon prediction results.**

| Sample Name | Location | Tn No. | Average Len (bp) |
| --- | --- | --- | --- |
| F130 | Chromosome | 33 | 591 |
|  | PlasmidA | 17 | 759 |
|  | PlasmidB | 1 | 464 |
| R124 | Chromosome | 3 | 438 |
| Z108 | Chromosome | 6 | 634 |
|  | PlasmidH | 1 | 377 |

**Supplementary Table S3. Plasmid comparison results.**

| Sample Name | Plasmid ID | Accession num | Seq length (bp) |
| --- | --- | --- | --- |
| F130 | PlasmidA | NZ_CP041264.2 | 230,007 |
| F130 | PlasmidB | NZ_KY662247.1 | 22,261 |
| F130 | PlasmidC | NZ_LR135231.1 | 7165 |
| F130 | PlasmidD | NZ_LR134113.1 | 4463 |
| F130 | PlasmidE | NZ_LR135202.1 | 3425 |
| F130 | PlasmidF | NZ_CP046076.1 | 1948 |
| F130 | PlasmidG | NC_002799.1 | 1928 |
| Z108 | PlasmidH | NC_006278.1 | 8104 |

**Supplementary Table S4. *Enterococcus faecium* pan-gene set containing strains.**

| No. | strains ID | No. | strains ID | No. | strains ID |
| --- | --- | --- | --- | --- | --- |
| 1 | GCA_016743855.1 | 95 | GCA_007923925.2 | 188 | GCA_030656835.1 |
| 2 | GCA_016864255.1 | 96 | GCA_024665675.1 | 189 | GCA_030656855.1 |
| 3 | GCA_017301355.1 | 97 | GCA_025800545.1 | 190 | GCA_030656935.1 |
| 4 | GCA_017584065.1 | 98 | GCA_025800645.1 | 191 | GCA_030657115.1 |
| 5 | GCA_017603725.1 | 99 | GCA_025800625.1 | 192 | GCA_030657135.1 |
| 6 | GCA_017815695.1 | 100 | GCA_025916235.1 | 193 | GCA_030657235.1 |
| 7 | GCA_017815655.1 | 101 | GCA_025913595.1 | 194 | GCA_030657275.1 |
| 8 | GCA_017815675.1 | 102 | GCA_026073355.1 | 195 | GCA_030656895.1 |
| 9 | GCA_017897965.1 | 103 | GCA_026073375.1 | 196 | GCA_030656915.1 |
| 10 | GCA_017898005.1 | 104 | GCA_026073475.1 | 197 | GCA_030656955.1 |
| 11 | GCA_017898025.1 | 105 | GCA_026073555.1 | 198 | GCA_030656975.1 |
| 12 | GCA_018219285.1 | 106 | GCA_026073435.1 | 199 | GCA_030664865.1 |
| 13 | GCA_018219325.1 | 107 | GCA_026073455.1 | 200 | GCA_030664905.1 |
| 14 | GCA_018279145.1 | 108 | GCA_026073535.1 | 201 | GCA_030664925.1 |
| 15 | GCA_907176135.1 | 109 | GCA_026073915.1 | 202 | GCA_030664985.1 |
| 16 | GCA_907176815.1 | 110 | GCA_023182835.2 | 203 | GCA_030665065.1 |
| 17 | GCA_907177285.1 | 111 | GCA_025998395.1 | 204 | GCA_030665125.1 |
| 18 | GCA_018516925.1 | 112 | GCA_026073115.1 | 205 | GCA_030665165.1 |
| 19 | GCA_018517045.1 | 113 | GCA_026073255.1 | 206 | GCA_030665285.1 |
| 20 | GCA_018517105.1 | 114 | GCA_026073415.1 | 207 | GCA_030685735.1 |
| 21 | GCA_018517145.1 | 115 | GCA_026073515.1 | 208 | GCA_030685835.1 |
| 22 | GCA_018516845.1 | 116 | GCA_026073625.1 | 209 | GCA_030685935.1 |
| 23 | GCA_018517025.1 | 117 | GCA_023182815.2 | 210 | GCA_030686035.1 |
| 24 | GCA_018517065.1 | 118 | GCA_007923905.2 | 211 | GCA_030664885.1 |
| 25 | GCA_018517165.1 | 119 | GCA_026073395.1 | 212 | GCA_030664945.1 |
| 26 | GCA_907165365.1 | 120 | GCA_026073495.1 | 213 | GCA_030665005.1 |
| 27 | GCA_018517185.1 | 121 | GCA_026073735.1 | 214 | GCA_030665225.1 |
| 28 | GCA_018517085.1 | 122 | GCA_026409165.1 | 215 | GCA_030665305.1 |
| 29 | GCA_019175445.1 | 123 | GCA_026427575.1 | 216 | GCA_030665365.1 |
| 30 | GCA_019175465.1 | 124 | GCA_027286245.1 | 217 | GCA_030665385.1 |
| 31 | GCA_019175425.1 | 125 | GCA_027915235.1 | 218 | GCA_030665245.1 |
| 32 | GCA_019175525.1 | 126 | GCA_027944535.1 | 219 | GCA_030665325.1 |
| 33 | GCA_019356355.1 | 127 | GCA_028335265.1 | 220 | GCA_030665405.1 |
| 34 | GCA_019456595.1 | 128 | GCA_028335465.1 | 221 | GCA_030685595.1 |
| 35 | GCA_907163255.1 | 129 | GCA_028335485.1 | 222 | GCA_030685675.1 |
| 36 | GCA_019456555.1 | 130 | GCA_028335565.1 | 223 | GCA_030685695.1 |
| 37 | GCA_019456575.1 | 131 | GCA_028335585.1 | 224 | GCA_030685775.1 |
| 38 | GCA_907163315.1 | 132 | GCA_028335245.1 | 225 | GCA_030685795.1 |
| 39 | GCA_019774555.1 | 133 | GCA_028335225.1 | 226 | GCA_030685895.1 |
| 40 | GCA_019977495.1 | 134 | GCA_028335525.1 | 227 | GCA_030685995.1 |
| 41 | GCA_019977575.1 | 135 | GCA_028335625.1 | 228 | GCA_030686095.1 |
| 42 | GCA_020162155.1 | 136 | GCA_028335205.1 | 229 | GCA_030685715.1 |
| 43 | GCA_020162175.1 | 137 | GCA_028335605.1 | 230 | GCA_030685815.1 |
| 44 | GCA_020091325.1 | 138 | GCA_020221735.2 | 231 | GCA_030685915.1 |
| 45 | GCA_020736625.1 | 139 | GCA_028891525.1 | 232 | GCA_030686015.1 |
| 46 | GCA_020736585.1 | 140 | GCA_029023785.1 | 233 | GCA_030656735.1 |
| 47 | GCA_021228615.1 | 141 | GCA_029167665.1 | 234 | GCA_030656795.1 |
| 48 | GCA_021398505.1 | 142 | GCA_007923965.2 | 235 | GCA_030656995.1 |
| 49 | GCA_021560195.1 | 143 | GCA_007923945.2 | 236 | GCA_030657035.1 |
| 50 | GCA_021713095.1 | 144 | GCA_007923785.2 | 237 | GCA_030657055.1 |
| 51 | GCA_021899455.1 | 145 | GCA_030064735.1 | 238 | GCA_030657175.1 |
| 52 | GCA_022648125.1 | 146 | GCA_007923865.2 | 239 | GCA_030657315.1 |
| 53 | GCA_022691445.1 | 147 | GCA_007923885.2 | 240 | GCA_030664805.1 |
| 54 | GCA_021172105.3 | 148 | GCA_030295785.1 | 241 | GCA_030664825.1 |
| 55 | GCA_022647825.1 | 149 | GCA_030316155.1 | 242 | GCA_030665025.1 |
| 56 | GCA_022699565.1 | 150 | GCA_030406065.1 | 243 | GCA_030665085.1 |
| 57 | GCA_022699225.1 | 151 | GCA_030581795.1 | 244 | GCA_030665145.1 |
| 58 | GCA_022699545.1 | 152 | GCA_030584225.1 | 245 | GCA_030665185.1 |
| 59 | GCA_022811165.1 | 153 | GCA_030584325.1 | 246 | GCA_030664785.1 |
| 60 | GCA_022811265.1 | 154 | GCA_030584425.1 | 247 | GCA_030664845.1 |
| 61 | GCA_022811365.1 | 155 | GCA_030584525.1 | 248 | GCA_030664965.1 |
| 62 | GCA_022811465.1 | 156 | GCA_030584625.1 | 249 | GCA_030665045.1 |
| 63 | GCA_022647945.1 | 157 | GCA_030581775.1 | 250 | GCA_030665105.1 |
| 64 | GCA_022811125.1 | 158 | GCA_030584205.1 | 251 | GCA_030665205.1 |
| 65 | GCA_022811145.1 | 159 | GCA_030584305.1 | 252 | GCA_030665265.1 |
| 66 | GCA_022811245.1 | 160 | GCA_030584405.1 | 253 | GCA_030665345.1 |
| 67 | GCA_022811345.1 | 161 | GCA_030584505.1 | 254 | GCA_030685655.1 |
| 68 | GCA_022811445.1 | 162 | GCA_030584605.1 | 255 | GCA_030685755.1 |
| 69 | GCA_022749455.1 | 163 | GCA_030581675.1 | 256 | GCA_030685855.1 |
| 70 | GCA_022811085.1 | 164 | GCA_030584265.1 | 257 | GCA_030685875.1 |
| 71 | GCA_022811185.1 | 165 | GCA_030584285.1 | 258 | GCA_030685955.1 |
| 72 | GCA_022811285.1 | 166 | GCA_030584365.1 | 259 | GCA_030685975.1 |
| 73 | GCA_022811385.1 | 167 | GCA_030584385.1 | 260 | GCA_030686055.1 |
| 74 | GCA_022811205.1 | 168 | GCA_030584465.1 | 261 | GCA_030686075.1 |
| 75 | GCA_022811225.1 | 169 | GCA_030584485.1 | 262 | GCA_030657155.1 |
| 76 | GCA_022811305.1 | 170 | GCA_030584585.1 | 263 | GCA_030657195.1 |
| 77 | GCA_022811325.1 | 171 | GCA_030584685.1 | 264 | GCA_030657255.1 |
| 78 | GCA_022811405.1 | 172 | GCA_030644805.1 | 265 | GCA_030656695.1 |
| 79 | GCA_022811425.1 | 173 | GCA_030644825.1 | 266 | GCA_030656755.1 |
| 80 | GCA_023204955.1 | 174 | GCA_030584245.1 | 267 | GCA_030656815.1 |
| 81 | GCA_023204975.1 | 175 | GCA_030584345.1 | 268 | GCA_030657015.1 |
| 82 | GCA_023299805.1 | 176 | GCA_030584445.1 | 269 | GCA_030657075.1 |
| 83 | GCA_023375345.1 | 177 | GCA_030584545.1 | 270 | GCA_030657095.1 |
| 84 | GCA_023375445.1 | 178 | GCA_030584645.1 | 271 | GCA_030719015.1 |
| 85 | GCA_023299325.1 | 179 | GCA_030584665.1 | 272 | GCA_033130085.1 |
| 86 | GCA_023299745.1 | 180 | GCA_030644745.1 | 273 | GCA_033247905.1 |
| 87 | GCA_023375425.1 | 181 | GCA_030644765.1 | 274 | GCA_033249005.1 |
| 88 | GCA_023299725.1 | 182 | GCA_030644785.1 | 275 | GCA_033260105.1 |
| 89 | GCA_023375205.1 | 183 | GCA_030657215.1 | 276 | GCA_033246985.1 |
| 90 | GCA_023375225.1 | 184 | GCA_030657295.1 | 277 | GCA_033249725.1 |
| 91 | GCA_023375405.1 | 185 | GCA_030643745.1 | 278 | GCA_033802685.1 |
| 92 | GCA_023375365.1 | 186 | GCA_030656715.1 | 279 | GCA_034644175.1 |
| 93 | GCA_023375385.1 | 187 | GCA_030656775.1 | 280 | F130 |
| 94 | GCA_023658035.1 |  |  |  |  |

**Supplementary Table S5. *Pediococcus pentosaceus* pan-gene set containing strains.**

| No. | strains ID | No. | strains ID | No. | strains ID |
| --- | --- | --- | --- | --- | --- |
| 1 | GCA_019008315.1 | 33 | GCA_022678665.1 | 65 | GCA_025132915.1 |
| 2 | GCA_016921135.1 | 34 | GCA_022394815.1 | 66 | GCA_025188065.1 |
| 3 | GCA_016726885.1 | 35 | GCA_020882545.2 | 67 | GCA_024622005.1 |
| 4 | GCA_016652195.1 | 36 | GCA_021378055.1 | 68 | GCA_024539715.1 |
| 5 | GCA_925299425.1 | 37 | GCA_021124455.1 | 69 | GCA_024580495.1 |
| 6 | GCA_023743315.1 | 38 | GCA_019793535.1 | 70 | GCA_024580845.1 |
| 7 | GCA_023743335.1 | 39 | GCA_019614475.1 | 71 | GCA_024349305.1 |
| 8 | GCA_023744355.1 | 40 | GCA_910579815.1 | 72 | GCA_033088855.1 |
| 9 | GCA_023744045.1 | 41 | GCA_023740675.1 | 73 | GCA_030984705.1 |
| 10 | GCA_925281455.1 | 42 | GCA_028067445.1 | 74 | GCA_030480445.1 |
| 11 | GCA_925289295.1 | 43 | GCA_027667435.1 | 75 | GCA_030433975.1 |
| 12 | GCA_023507505.1 | 44 | GCA_027688855.1 | 76 | GCA_949118755.1 |
| 13 | GCA_023277805.1 | 45 | GCA_938029495.1 | 77 | GCA_030403405.1 |
| 14 | GCA_023369775.1 | 46 | GCA_027675905.1 | 78 | GCA_949483295.1 |
| 15 | GCA_023277785.1 | 47 | GCA_027693945.1 | 79 | GCA_030291815.1 |
| 16 | GCA_023277765.1 | 48 | GCA_026226215.1 | 80 | GCA_030291835.1 |
| 17 | GCA_022690785.1 | 49 | GCA_025770495.1 | 81 | GCA_030291855.1 |
| 18 | GCA_022642265.1 | 50 | GCA_025770455.1 | 82 | GCA_949483205.1 |
| 19 | GCA_022645105.1 | 51 | GCA_025770465.1 | 83 | GCA_029823395.1 |
| 20 | GCA_022645325.1 | 52 | GCA_025122115.1 | 84 | GCA_029439595.1 |
| 21 | GCA_022646465.1 | 53 | GCA_025122195.1 | 85 | GCA_029542265.1 |
| 22 | GCA_022483435.1 | 54 | GCA_025122025.1 | 86 | GCA_029070885.1 |
| 23 | GCA_022483125.1 | 55 | GCA_025122095.1 | 87 | GCA_028994255.1 |
| 24 | GCA_022484085.1 | 56 | GCA_025122175.1 | 88 | GCA_028888655.1 |
| 25 | GCA_022483705.1 | 57 | GCA_025132875.1 | 89 | GCA_028656295.1 |
| 26 | GCA_022644945.1 | 58 | GCA_025122155.1 | 90 | GCA_028656215.1 |
| 27 | GCA_022646925.1 | 59 | GCA_025188145.1 | 91 | GCA_028206435.1 |
| 28 | GCA_022641855.1 | 60 | GCA_025122005.1 | 92 | GCA_035209715.1 |
| 29 | GCA_022641845.1 | 61 | GCA_025122105.1 | 93 | GCA_035321745.1 |
| 30 | GCA_022642915.1 | 62 | GCA_025188055.1 | 94 | GCA_034321905.1 |
| 31 | GCA_022643425.1 | 63 | GCA_025188105.1 | 95 | GCA_033882205.1 |
| 32 | GCA_022646785.1 | 64 | GCA_025188125.1 | 96 | R124 |

**Supplementary Table S6. *Lactiplantibacillus plantarum* pan-gene set containing strains.**

| No. | strains ID | No. | strains ID | No. | strains ID |
| --- | --- | --- | --- | --- | --- |
| 1 | GCA_000466905.3 | 43 | GCA_027474465.1 | 85 | GCA_031348585.1 |
| 2 | GCA_019076805.1 | 44 | GCA_027558615.1 | 86 | GCA_030758995.1 |
| 3 | GCA_018351295.1 | 45 | GCA_026976315.1 | 87 | GCA_011170185.2 |
| 4 | GCA_017742875.1 | 46 | GCA_026689375.1 | 88 | GCA_030503695.1 |
| 5 | GCA_017798305.1 | 47 | GCA_026183415.1 | 89 | GCA_030463605.1 |
| 6 | GCA_017576965.1 | 48 | GCA_026016545.1 | 90 | GCA_030406025.1 |
| 7 | GCA_017301935.1 | 49 | GCA_026153115.1 | 91 | GCA_030297715.1 |
| 8 | GCA_017351995.1 | 50 | GCA_026240755.1 | 92 | GCA_030297735.1 |
| 9 | GCA_017068235.1 | 51 | GCA_026013765.1 | 93 | GCA_030253605.1 |
| 10 | GCA_017068215.1 | 52 | GCA_025723165.1 | 94 | GCA_030297695.1 |
| 11 | GCA_016812075.1 | 53 | GCA_025402835.1 | 95 | GCA_030061985.1 |
| 12 | GCA_016894405.1 | 54 | GCA_024969715.1 | 96 | GCA_030061955.1 |
| 13 | GCA_016775685.1 | 55 | GCA_024969905.1 | 97 | GCA_030061915.1 |
| 14 | GCA_016838645.1 | 56 | GCA_025144505.1 | 98 | GCA_030061935.1 |
| 15 | GCA_016598735.1 | 57 | GCA_024732385.1 | 99 | GCA_029834415.1 |
| 16 | GCA_023278325.1 | 58 | GCA_024758745.1 | 100 | GCA_029854235.1 |
| 17 | GCA_011304595.2 | 59 | GCA_024970165.1 | 101 | GCA_029854335.1 |
| 18 | GCA_023370155.1 | 60 | GCA_024970145.1 | 102 | GCA_029814785.1 |
| 19 | GCA_023348385.1 | 61 | GCA_024970125.1 | 103 | GCA_029854315.1 |
| 20 | GCA_023348465.1 | 62 | GCA_024800605.1 | 104 | GCA_029855105.1 |
| 21 | GCA_023348525.1 | 63 | GCA_024758665.1 | 105 | GCA_029906425.1 |
| 22 | GCA_023207995.1 | 64 | GCA_024442115.1 | 106 | GCA_029543005.1 |
| 23 | GCA_023347215.1 | 65 | GCA_024396815.1 | 107 | GCA_029590535.1 |
| 24 | GCA_018588615.2 | 66 | GCA_024137985.1 | 108 | GCA_029637825.1 |
| 25 | GCA_021279005.2 | 67 | GCA_024181685.1 | 109 | GCA_029542245.1 |
| 26 | GCA_018588665.2 | 68 | GCA_024181705.1 | 110 | GCA_029537295.1 |
| 27 | GCA_022810685.1 | 69 | GCA_024137845.1 | 111 | GCA_028768485.1 |
| 28 | GCA_018588605.2 | 70 | GCA_023973045.1 | 112 | GCA_028869445.1 |
| 29 | GCA_022558425.1 | 71 | GCA_028201575.1 | 113 | GCA_028411375.1 |
| 30 | GCA_021559915.1 | 72 | GCA_033024555.1 | 114 | GCA_028463965.1 |
| 31 | GCA_021559675.1 | 73 | GCA_032818175.1 | 115 | GCA_035338015.1 |
| 32 | GCA_021560135.1 | 74 | GCA_032920365.1 | 116 | GCA_035588615.1 |
| 33 | GCA_021650875.1 | 75 | GCA_032921125.1 | 117 | GCA_035336205.1 |
| 34 | GCA_020881935.1 | 76 | GCA_032602185.1 | 118 | GCA_035328725.1 |
| 35 | GCA_019425695.1 | 77 | GCA_032463565.1 | 119 | GCA_034479655.1 |
| 36 | GCA_019321805.1 | 78 | GCA_032463585.1 | 120 | GCA_034333585.1 |
| 37 | GCA_019399915.1 | 79 | GCA_031876755.1 | 121 | GCA_034422915.1 |
| 38 | GCA_019469465.1 | 80 | GCA_031877725.1 | 122 | GCA_034426955.1 |
| 39 | GCA_019211785.1 | 81 | GCA_031597175.1 | 123 | GCA_034478365.1 |
| 40 | GCA_019211765.1 | 82 | GCA_031583165.1 | 124 | GCA_033802745.1 |
| 41 | GCA_027920405.1 | 83 | GCA_031432955.1 | 125 | GCA_033802805.1 |
| 42 | GCA_027557615.1 | 84 | GCA_031596315.1 | 126 | GCA_033546835.1 |
|  |  |  |  | 127 | Z108 |

**Supplementary Table S9. *Enterococcus faecium* pan-genes mobileOG prediction results.**

| ID | RRR | P | T | STD | IE | ID | RRR | P | T | STD | IE |  |
| --- | --- | --- | --- | --- | --- | --- | --- | --- | --- | --- | --- | --- |
| F130 | 80 | 58 | 38 | 30 | 94 | GCA_029023785.1 | 62 | 30 | 24 | 17 | 46 | |
| GCA_007923785.2 | 61 | 37 | 28 | 7 | 43 | GCA_029167665.1 | 73 | 43 | 34 | 28 | 117 | |
| GCA_007923865.2 | 64 | 18 | 24 | 10 | 47 | GCA_030064735.1 | 69 | 37 | 30 | 11 | 41 | |
| GCA_007923885.2 | 61 | 47 | 23 | 12 | 47 | GCA_030295785.1 | 65 | 45 | 34 | 24 | 71 | |
| GCA_007923905.2 | 64 | 32 | 23 | 14 | 43 | GCA_030316155.1 | 65 | 48 | 26 | 18 | 72 | |
| GCA_007923925.2 | 62 | 37 | 24 | 9 | 39 | GCA_030406065.1 | 60 | 50 | 24 | 17 | 56 | |
| GCA_007923945.2 | 64 | 41 | 24 | 9 | 42 | GCA_030581675.1 | 68 | 49 | 23 | 20 | 69 | |
| GCA_007923965.2 | 63 | 47 | 25 | 11 | 43 | GCA_030581775.1 | 65 | 35 | 29 | 16 | 65 | |
| GCA_016743855.1 | 71 | 48 | 34 | 20 | 68 | GCA_030581795.1 | 63 | 34 | 23 | 15 | 50 | |
| GCA_016864255.1 | 72 | 63 | 33 | 23 | 91 | GCA_030584205.1 | 67 | 43 | 24 | 15 | 65 | |
| GCA_017301355.1 | 76 | 48 | 36 | 40 | 132 | GCA_030584225.1 | 67 | 44 | 26 | 17 | 66 | |
| GCA_017584065.1 | 74 | 35 | 43 | 24 | 117 | GCA_030584245.1 | 68 | 47 | 27 | 18 | 61 | |
| GCA_017603725.1 | 72 | 40 | 32 | 36 | 128 | GCA_030584265.1 | 63 | 40 | 24 | 17 | 67 | |
| GCA_017815655.1 | 69 | 49 | 27 | 30 | 137 | GCA_030584285.1 | 69 | 58 | 26 | 24 | 83 | |
| GCA_017815675.1 | 76 | 42 | 33 | 25 | 97 | GCA_030584305.1 | 66 | 49 | 26 | 18 | 75 | |
| GCA_017815695.1 | 73 | 43 | 32 | 36 | 119 | GCA_030584325.1 | 63 | 34 | 25 | 14 | 57 | |
| GCA_017897965.1 | 80 | 44 | 33 | 27 | 103 | GCA_030584345.1 | 68 | 43 | 25 | 19 | 70 | |
| GCA_017898005.1 | 80 | 44 | 33 | 27 | 103 | GCA_030584365.1 | 69 | 43 | 26 | 21 | 78 | |
| GCA_017898025.1 | 83 | 43 | 37 | 38 | 114 | GCA_030584385.1 | 66 | 48 | 26 | 16 | 72 | |
| GCA_018219285.1 | 71 | 72 | 29 | 18 | 77 | GCA_030584405.1 | 70 | 43 | 26 | 21 | 77 | |
| GCA_018219325.1 | 76 | 50 | 46 | 24 | 120 | GCA_030584425.1 | 67 | 43 | 26 | 21 | 78 | |
| GCA_018279145.1 | 67 | 45 | 33 | 23 | 51 | GCA_030584445.1 | 64 | 48 | 24 | 15 | 71 | |
| GCA_018516845.1 | 60 | 33 | 28 | 8 | 30 | GCA_030584465.1 | 66 | 35 | 29 | 16 | 67 | |
| GCA_018516925.1 | 75 | 42 | 27 | 20 | 85 | GCA_030584485.1 | 68 | 48 | 26 | 22 | 77 | |
| GCA_018517025.1 | 68 | 39 | 35 | 21 | 60 | GCA_030584505.1 | 66 | 49 | 26 | 22 | 79 | |
| GCA_018517045.1 | 67 | 39 | 34 | 21 | 53 | GCA_030584525.1 | 62 | 33 | 24 | 13 | 53 | |
| GCA_018517065.1 | 65 | 45 | 34 | 21 | 65 | GCA_030584545.1 | 66 | 49 | 26 | 22 | 79 | |
| GCA_018517085.1 | 67 | 45 | 36 | 16 | 49 | GCA_030584585.1 | 67 | 40 | 26 | 20 | 80 | |
| GCA_018517105.1 | 66 | 42 | 46 | 27 | 69 | GCA_030584605.1 | 67 | 40 | 26 | 20 | 82 | |
| GCA_018517145.1 | 74 | 39 | 38 | 18 | 94 | GCA_030584625.1 | 68 | 49 | 26 | 22 | 80 | |
| GCA_018517165.1 | 66 | 38 | 33 | 18 | 76 | GCA_030584645.1 | 68 | 39 | 26 | 27 | 91 | |
| GCA_018517185.1 | 64 | 45 | 24 | 12 | 47 | GCA_030584665.1 | 68 | 42 | 26 | 21 | 71 | |
| GCA_019175425.1 | 65 | 44 | 34 | 24 | 69 | GCA_030584685.1 | 66 | 49 | 26 | 22 | 78 | |
| GCA_019175445.1 | 63 | 28 | 25 | 20 | 53 | GCA_030643745.1 | 56 | 32 | 16 | 6 | 18 | |
| GCA_019175465.1 | 70 | 31 | 35 | 25 | 55 | GCA_030644745.1 | 66 | 49 | 26 | 22 | 80 | |
| GCA_019175525.1 | 65 | 31 | 28 | 16 | 33 | GCA_030644765.1 | 69 | 49 | 26 | 22 | 77 | |
| GCA_019356355.1 | 65 | 34 | 27 | 13 | 59 | GCA_030644785.1 | 66 | 49 | 26 | 22 | 80 | |
| GCA_019456555.1 | 62 | 18 | 30 | 15 | 57 | GCA_030644805.1 | 67 | 48 | 27 | 18 | 74 | |
| GCA_019456575.1 | 62 | 18 | 30 | 15 | 57 | GCA_030644825.1 | 68 | 48 | 27 | 18 | 71 | |
| GCA_019456595.1 | 62 | 18 | 30 | 15 | 56 | GCA_030656695.1 | 67 | 40 | 26 | 20 | 87 | |
| GCA_019774555.1 | 69 | 20 | 34 | 25 | 96 | GCA_030656715.1 | 62 | 50 | 23 | 17 | 69 | |
| GCA_019977495.1 | 73 | 41 | 40 | 23 | 110 | GCA_030656735.1 | 67 | 38 | 25 | 18 | 67 | |
| GCA_019977575.1 | 76 | 41 | 41 | 28 | 123 | GCA_030656755.1 | 66 | 44 | 26 | 16 | 66 | |
| GCA_020091325.1 | 70 | 41 | 27 | 30 | 100 | GCA_030656775.1 | 66 | 48 | 26 | 17 | 64 | |
| GCA_020162155.1 | 73 | 40 | 39 | 29 | 129 | GCA_030656795.1 | 68 | 42 | 27 | 21 | 69 | |
| GCA_020162175.1 | 73 | 40 | 39 | 29 | 115 | GCA_030656815.1 | 67 | 49 | 26 | 22 | 79 | |
| GCA_020221735.2 | 74 | 21 | 39 | 23 | 111 | GCA_030656835.1 | 67 | 40 | 26 | 20 | 79 | |
| GCA_020736585.1 | 80 | 41 | 44 | 35 | 103 | GCA_030656855.1 | 73 | 50 | 30 | 15 | 57 | |
| GCA_020736625.1 | 80 | 41 | 44 | 35 | 110 | GCA_030656895.1 | 66 | 50 | 26 | 19 | 72 | |
| GCA_021172105.3 | 75 | 49 | 33 | 36 | 118 | GCA_030656915.1 | 68 | 43 | 26 | 15 | 61 | |
| GCA_021228615.1 | 78 | 48 | 43 | 23 | 111 | GCA_030656935.1 | 61 | 25 | 23 | 16 | 55 | |
| GCA_021398505.1 | 60 | 27 | 22 | 11 | 27 | GCA_030656955.1 | 66 | 50 | 26 | 19 | 73 | |
| GCA_021560195.1 | 67 | 43 | 37 | 25 | 82 | GCA_030656975.1 | 67 | 48 | 27 | 18 | 74 | |
| GCA_021713095.1 | 76 | 64 | 38 | 27 | 99 | GCA_030656995.1 | 62 | 37 | 22 | 16 | 65 | |
| GCA_021899455.1 | 67 | 41 | 34 | 27 | 119 | GCA_030657015.1 | 64 | 48 | 26 | 15 | 63 | |
| GCA_022647825.1 | 78 | 42 | 38 | 27 | 110 | GCA_030657035.1 | 64 | 48 | 24 | 16 | 69 | |
| GCA_022647945.1 | 78 | 42 | 37 | 27 | 107 | GCA_030657055.1 | 68 | 49 | 26 | 22 | 73 | |
| GCA_022648125.1 | 78 | 42 | 37 | 27 | 110 | GCA_030657075.1 | 68 | 48 | 26 | 23 | 76 | |
| GCA_022691445.1 | 71 | 27 | 35 | 22 | 121 | GCA_030657095.1 | 68 | 44 | 29 | 15 | 69 | |
| GCA_022699225.1 | 75 | 51 | 36 | 21 | 105 | GCA_030657115.1 | 67 | 51 | 27 | 18 | 70 | |
| GCA_022699545.1 | 69 | 42 | 27 | 30 | 113 | GCA_030657135.1 | 64 | 36 | 21 | 17 | 69 | |
| GCA_022699565.1 | 71 | 42 | 31 | 28 | 105 | GCA_030657155.1 | 68 | 45 | 26 | 15 | 67 | |
| GCA_022749455.1 | 69 | 51 | 40 | 35 | 105 | GCA_030657175.1 | 67 | 40 | 26 | 21 | 81 | |
| GCA_022811085.1 | 75 | 46 | 42 | 39 | 125 | GCA_030657195.1 | 64 | 43 | 26 | 15 | 65 | |
| GCA_022811125.1 | 75 | 49 | 35 | 29 | 97 | GCA_030657215.1 | 70 | 40 | 26 | 21 | 83 | |
| GCA_022811145.1 | 82 | 41 | 43 | 27 | 114 | GCA_030657235.1 | 66 | 37 | 26 | 15 | 67 | |
| GCA_022811165.1 | 71 | 33 | 39 | 35 | 128 | GCA_030657255.1 | 68 | 49 | 26 | 22 | 77 | |
| GCA_022811185.1 | 77 | 43 | 37 | 29 | 97 | GCA_030657275.1 | 66 | 38 | 26 | 22 | 77 | |
| GCA_022811205.1 | 77 | 76 | 29 | 31 | 103 | GCA_030657295.1 | 62 | 48 | 19 | 15 | 67 | |
| GCA_022811225.1 | 71 | 66 | 37 | 38 | 117 | GCA_030657315.1 | 65 | 52 | 26 | 17 | 71 | |
| GCA_022811245.1 | 75 | 49 | 35 | 29 | 96 | GCA_030664785.1 | 65 | 38 | 23 | 12 | 60 | |
| GCA_022811265.1 | 84 | 41 | 43 | 27 | 111 | GCA_030664805.1 | 67 | 35 | 29 | 15 | 67 | |
| GCA_022811285.1 | 77 | 43 | 37 | 29 | 98 | GCA_030664825.1 | 67 | 37 | 26 | 25 | 103 | |
| GCA_022811305.1 | 72 | 39 | 44 | 32 | 116 | GCA_030664845.1 | 66 | 48 | 26 | 15 | 69 | |
| GCA_022811325.1 | 74 | 50 | 30 | 24 | 113 | GCA_030664865.1 | 64 | 49 | 24 | 21 | 68 | |
| GCA_022811345.1 | 76 | 50 | 32 | 22 | 114 | GCA_030664885.1 | 69 | 49 | 26 | 23 | 80 | |
| GCA_022811365.1 | 71 | 63 | 38 | 38 | 116 | GCA_030664905.1 | 66 | 50 | 26 | 18 | 68 | |
| GCA_022811385.1 | 79 | 76 | 29 | 31 | 95 | GCA_030664925.1 | 63 | 38 | 22 | 12 | 64 | |
| GCA_022811405.1 | 79 | 42 | 35 | 29 | 103 | GCA_030664945.1 | 67 | 49 | 26 | 23 | 83 | |
| GCA_022811425.1 | 78 | 76 | 29 | 30 | 94 | GCA_030664965.1 | 66 | 49 | 28 | 18 | 76 | |
| GCA_022811445.1 | 77 | 63 | 30 | 30 | 92 | GCA_030664985.1 | 66 | 35 | 29 | 15 | 67 | |
| GCA_022811465.1 | 80 | 41 | 42 | 24 | 100 | GCA_030665005.1 | 66 | 48 | 23 | 12 | 65 | |
| GCA_023182815.2 | 69 | 30 | 36 | 24 | 70 | GCA_030665025.1 | 66 | 47 | 23 | 10 | 65 | |
| GCA_023182835.2 | 65 | 52 | 34 | 26 | 91 | GCA_030665045.1 | 67 | 49 | 26 | 21 | 82 | |
| GCA_023204955.1 | 80 | 43 | 40 | 34 | 125 | GCA_030665065.1 | 67 | 35 | 29 | 15 | 67 | |
| GCA_023204975.1 | 79 | 43 | 41 | 25 | 102 | GCA_030665085.1 | 67 | 31 | 26 | 22 | 75 | |
| GCA_023299325.1 | 65 | 34 | 38 | 26 | 74 | GCA_030665105.1 | 68 | 49 | 24 | 22 | 72 | |
| GCA_023299725.1 | 71 | 39 | 41 | 23 | 57 | GCA_030665125.1 | 67 | 43 | 29 | 16 | 71 | |
| GCA_023299745.1 | 71 | 54 | 36 | 18 | 61 | GCA_030665145.1 | 64 | 43 | 24 | 14 | 74 | |
| GCA_023299805.1 | 76 | 42 | 37 | 11 | 49 | GCA_030665165.1 | 67 | 44 | 26 | 15 | 66 | |
| GCA_023375205.1 | 62 | 20 | 29 | 20 | 60 | GCA_030665185.1 | 66 | 44 | 29 | 15 | 69 | |
| GCA_023375225.1 | 65 | 39 | 26 | 8 | 30 | GCA_030665205.1 | 66 | 49 | 26 | 18 | 70 | |
| GCA_023375345.1 | 66 | 18 | 34 | 21 | 55 | GCA_030665225.1 | 67 | 38 | 26 | 22 | 75 | |
| GCA_023375365.1 | 61 | 30 | 26 | 12 | 45 | GCA_030665245.1 | 66 | 49 | 26 | 18 | 74 | |
| GCA_023375385.1 | 68 | 29 | 31 | 19 | 55 | GCA_030665265.1 | 67 | 43 | 29 | 16 | 72 | |
| GCA_023375405.1 | 60 | 18 | 33 | 20 | 62 | GCA_030665285.1 | 68 | 47 | 26 | 15 | 65 | |
| GCA_023375425.1 | 63 | 44 | 26 | 21 | 53 | GCA_030665305.1 | 74 | 37 | 30 | 26 | 83 | |
| GCA_023375445.1 | 65 | 40 | 26 | 10 | 39 | GCA_030665325.1 | 68 | 47 | 26 | 15 | 65 | |
| GCA_023658035.1 | 74 | 49 | 29 | 29 | 102 | GCA_030665345.1 | 68 | 44 | 29 | 16 | 69 | |
| GCA_024665675.1 | 62 | 44 | 18 | 6 | 24 | GCA_030665365.1 | 66 | 50 | 25 | 20 | 73 | |
| GCA_025800545.1 | 63 | 31 | 32 | 13 | 51 | GCA_030665385.1 | 72 | 45 | 30 | 21 | 69 | |
| GCA_025800625.1 | 67 | 43 | 31 | 22 | 65 | GCA_030665405.1 | 73 | 46 | 31 | 22 | 71 | |
| GCA_025800645.1 | 67 | 33 | 31 | 21 | 62 | GCA_030685595.1 | 68 | 49 | 26 | 23 | 76 | |
| GCA_025913595.1 | 65 | 40 | 31 | 24 | 75 | GCA_030685655.1 | 64 | 49 | 20 | 20 | 77 | |
| GCA_025916235.1 | 71 | 38 | 40 | 29 | 77 | GCA_030685675.1 | 68 | 49 | 26 | 22 | 77 | |
| GCA_025998395.1 | 83 | 37 | 37 | 38 | 130 | GCA_030685695.1 | 70 | 49 | 30 | 40 | 127 | |
| GCA_026073115.1 | 80 | 69 | 44 | 33 | 140 | GCA_030685715.1 | 69 | 49 | 28 | 22 | 71 | |
| GCA_026073255.1 | 80 | 69 | 45 | 31 | 129 | GCA_030685735.1 | 66 | 38 | 24 | 15 | 63 | |
| GCA_026073355.1 | 81 | 47 | 47 | 34 | 139 | GCA_030685755.1 | 64 | 48 | 26 | 22 | 76 | |
| GCA_026073375.1 | 78 | 47 | 49 | 32 | 142 | GCA_030685775.1 | 68 | 50 | 24 | 23 | 70 | |
| GCA_026073395.1 | 81 | 46 | 47 | 34 | 142 | GCA_030685795.1 | 64 | 49 | 28 | 22 | 81 | |
| GCA_026073415.1 | 70 | 85 | 27 | 20 | 76 | GCA_030685815.1 | 65 | 38 | 24 | 16 | 65 | |
| GCA_026073435.1 | 76 | 34 | 36 | 34 | 122 | GCA_030685835.1 | 61 | 35 | 23 | 13 | 67 | |
| GCA_026073455.1 | 75 | 54 | 45 | 36 | 147 | GCA_030685855.1 | 70 | 48 | 26 | 23 | 73 | |
| GCA_026073475.1 | 83 | 44 | 45 | 37 | 138 | GCA_030685875.1 | 67 | 46 | 22 | 19 | 81 | |
| GCA_026073495.1 | 75 | 64 | 34 | 27 | 113 | GCA_030685895.1 | 60 | 46 | 23 | 14 | 73 | |
| GCA_026073515.1 | 73 | 47 | 35 | 30 | 141 | GCA_030685915.1 | 62 | 34 | 22 | 16 | 71 | |
| GCA_026073535.1 | 77 | 59 | 41 | 33 | 130 | GCA_030685935.1 | 68 | 49 | 26 | 22 | 78 | |
| GCA_026073555.1 | 80 | 39 | 49 | 39 | 149 | GCA_030685955.1 | 66 | 49 | 25 | 19 | 75 | |
| GCA_026073625.1 | 74 | 54 | 34 | 27 | 97 | GCA_030685975.1 | 68 | 49 | 28 | 21 | 74 | |
| GCA_026073735.1 | 74 | 53 | 35 | 28 | 96 | GCA_030685995.1 | 66 | 48 | 26 | 18 | 70 | |
| GCA_026073915.1 | 74 | 54 | 33 | 28 | 101 | GCA_030686015.1 | 68 | 49 | 26 | 22 | 78 | |
| GCA_026409165.1 | 77 | 63 | 39 | 32 | 105 | GCA_030686035.1 | 68 | 49 | 26 | 22 | 77 | |
| GCA_026427575.1 | 58 | 20 | 19 | 14 | 41 | GCA_030686055.1 | 66 | 38 | 23 | 16 | 63 | |
| GCA_027286245.1 | 72 | 58 | 30 | 28 | 103 | GCA_030686075.1 | 65 | 50 | 22 | 18 | 68 | |
| GCA_027915235.1 | 56 | 31 | 16 | 6 | 17 | GCA_030686095.1 | 66 | 41 | 26 | 22 | 81 | |
| GCA_027944535.1 | 61 | 17 | 32 | 17 | 79 | GCA_030719015.1 | 68 | 42 | 25 | 10 | 30 | |
| GCA_028335205.1 | 68 | 29 | 29 | 21 | 54 | GCA_033130085.1 | 73 | 40 | 42 | 27 | 88 | |
| GCA_028335225.1 | 67 | 38 | 33 | 19 | 53 | GCA_033246985.1 | 66 | 30 | 29 | 13 | 68 | |
| GCA_028335245.1 | 66 | 31 | 37 | 15 | 66 | GCA_033247905.1 | 62 | 17 | 32 | 14 | 52 | |
| GCA_028335265.1 | 70 | 28 | 33 | 18 | 62 | GCA_033249005.1 | 67 | 29 | 30 | 17 | 72 | |
| GCA_028335465.1 | 71 | 40 | 36 | 21 | 58 | GCA_033249725.1 | 68 | 17 | 31 | 18 | 64 | |
| GCA_028335485.1 | 67 | 37 | 32 | 19 | 52 | GCA_033260105.1 | 64 | 16 | 30 | 13 | 71 | |
| GCA_028335525.1 | 68 | 43 | 28 | 15 | 36 | GCA_033802685.1 | 60 | 45 | 28 | 24 | 58 | |
| GCA_028335565.1 | 63 | 40 | 36 | 18 | 68 | GCA_034644175.1 | 63 | 49 | 25 | 14 | 32 | |
| GCA_028335585.1 | 64 | 41 | 39 | 27 | 85 | GCA_907163255.1 | 78 | 43 | 33 | 24 | 107 | |
| GCA_028335605.1 | 63 | 40 | 36 | 18 | 71 | GCA_907163315.1 | 78 | 43 | 33 | 24 | 111 | |
| GCA_028335625.1 | 66 | 40 | 41 | 22 | 73 | GCA_907165365.1 | 79 | 50 | 31 | 28 | 102 | |
| GCA_028891525.1 | 68 | 42 | 34 | 29 | 69 | GCA_907176135.1 | 73 | 47 | 32 | 25 | 102 | |
| GCA_907177285.1 | 78 | 47 | 34 | 29 | 93 | GCA_907176815.1 | 73 | 47 | 32 | 25 | 96 | |

**Supplementary Table S10. *Enterococcus faecium* pan-genes PlamidFinder prediction results.**

| ID | No. | ID | No. | ID | No. |
| --- | --- | --- | --- | --- | --- |
| F130 | 7 | GCA_023299805.1 | 6 | GCA_030656755.1 | 1 |
| GCA_007923785.2 | 1 | GCA_023375205.1 | 3 | GCA_030656775.1 | 1 |
| GCA_007923865.2 | 1 | GCA_023375225.1 | 2 | GCA_030656795.1 | 2 |
| GCA_007923885.2 | 1 | GCA_023375345.1 | 3 | GCA_030656815.1 | 1 |
| GCA_007923905.2 | 1 | GCA_023375365.1 | 2 | GCA_030656835.1 | 1 |
| GCA_007923925.2 | 1 | GCA_023375385.1 | 4 | GCA_030656855.1 | 2 |
| GCA_007923945.2 | 1 | GCA_023375405.1 | 3 | GCA_030656895.1 | 1 |
| GCA_007923965.2 | 1 | GCA_023375425.1 | 2 | GCA_030656915.1 | 1 |
| GCA_016743855.1 | 4 | GCA_023375445.1 | 3 | GCA_030656955.1 | 1 |
| GCA_016864255.1 | 3 | GCA_023658035.1 | 8 | GCA_030656975.1 | 1 |
| GCA_017301355.1 | 4 | GCA_024665675.1 | 1 | GCA_030656995.1 | 2 |
| GCA_017584065.1 | 8 | GCA_025800545.1 | 3 | GCA_030657015.1 | 1 |
| GCA_017603725.1 | 8 | GCA_025800625.1 | 4 | GCA_030657035.1 | 1 |
| GCA_017815655.1 | 4 | GCA_025800645.1 | 3 | GCA_030657055.1 | 1 |
| GCA_017815675.1 | 10 | GCA_025913595.1 | 2 | GCA_030657075.1 | 1 |
| GCA_017815695.1 | 7 | GCA_025916235.1 | 4 | GCA_030657095.1 | 1 |
| GCA_017897965.1 | 6 | GCA_025998395.1 | 7 | GCA_030657115.1 | 1 |
| GCA_017898005.1 | 6 | GCA_026073115.1 | 6 | GCA_030657155.1 | 1 |
| GCA_017898025.1 | 9 | GCA_026073255.1 | 5 | GCA_030657175.1 | 1 |
| GCA_018219285.1 | 3 | GCA_026073355.1 | 9 | GCA_030657195.1 | 1 |
| GCA_018219325.1 | 7 | GCA_026073375.1 | 11 | GCA_030657215.1 | 1 |
| GCA_018279145.1 | 3 | GCA_026073395.1 | 11 | GCA_030657235.1 | 1 |
| GCA_018516845.1 | 2 | GCA_026073415.1 | 2 | GCA_030657255.1 | 1 |
| GCA_018516925.1 | 7 | GCA_026073435.1 | 4 | GCA_030657275.1 | 2 |
| GCA_018517025.1 | 4 | GCA_026073455.1 | 4 | GCA_030657295.1 | 2 |
| GCA_018517045.1 | 4 | GCA_026073475.1 | 6 | GCA_030657315.1 | 1 |
| GCA_018517065.1 | 3 | GCA_026073495.1 | 1 | GCA_030664785.1 | 1 |
| GCA_018517085.1 | 5 | GCA_026073515.1 | 4 | GCA_030664805.1 | 1 |
| GCA_018517105.1 | 5 | GCA_026073535.1 | 5 | GCA_030664825.1 | 2 |
| GCA_018517145.1 | 5 | GCA_026073555.1 | 6 | GCA_030664845.1 | 1 |
| GCA_018517165.1 | 3 | GCA_026073625.1 | 4 | GCA_030664865.1 | 1 |
| GCA_018517185.1 | 2 | GCA_026073735.1 | 4 | GCA_030664885.1 | 1 |
| GCA_019175425.1 | 3 | GCA_026073915.1 | 3 | GCA_030664905.1 | 2 |
| GCA_019175445.1 | 1 | GCA_026409165.1 | 5 | GCA_030664925.1 | 1 |
| GCA_019175465.1 | 5 | GCA_026427575.1 | 2 | GCA_030664945.1 | 1 |
| GCA_019175525.1 | 1 | GCA_027286245.1 | 5 | GCA_030664965.1 | 2 |
| GCA_019356355.1 | 2 | GCA_027944535.1 | 3 | GCA_030664985.1 | 1 |
| GCA_019456555.1 | 2 | GCA_028335205.1 | 3 | GCA_030665005.1 | 1 |
| GCA_019456575.1 | 2 | GCA_028335225.1 | 5 | GCA_030665025.1 | 1 |
| GCA_019456595.1 | 2 | GCA_028335245.1 | 3 | GCA_030665045.1 | 1 |
| GCA_019774555.1 | 4 | GCA_028335265.1 | 3 | GCA_030665065.1 | 1 |
| GCA_019977495.1 | 6 | GCA_028335465.1 | 4 | GCA_030665085.1 | 1 |
| GCA_019977575.1 | 7 | GCA_028335485.1 | 5 | GCA_030665105.1 | 1 |
| GCA_020091325.1 | 3 | GCA_028335525.1 | 3 | GCA_030665125.1 | 1 |
| GCA_020162155.1 | 8 | GCA_028335565.1 | 2 | GCA_030665145.1 | 1 |
| GCA_020162175.1 | 7 | GCA_028335585.1 | 3 | GCA_030665165.1 | 1 |
| GCA_020221735.2 | 7 | GCA_028335605.1 | 2 | GCA_030665185.1 | 2 |
| GCA_020736585.1 | 7 | GCA_028335625.1 | 2 | GCA_030665205.1 | 1 |
| GCA_020736625.1 | 7 | GCA_028891525.1 | 3 | GCA_030665225.1 | 1 |
| GCA_021172105.3 | 6 | GCA_029023785.1 | 1 | GCA_030665245.1 | 1 |
| GCA_021228615.1 | 5 | GCA_029167665.1 | 7 | GCA_030665265.1 | 1 |
| GCA_021398505.1 | 1 | GCA_030064735.1 | 4 | GCA_030665285.1 | 1 |
| GCA_021560195.1 | 4 | GCA_030295785.1 | 3 | GCA_030665305.1 | 2 |
| GCA_021713095.1 | 4 | GCA_030316155.1 | 1 | GCA_030665325.1 | 1 |
| GCA_021899455.1 | 4 | GCA_030406065.1 | 1 | GCA_030665345.1 | 1 |
| GCA_022647825.1 | 4 | GCA_030581675.1 | 1 | GCA_030665405.1 | 5 |
| GCA_022647945.1 | 4 | GCA_030581775.1 | 1 | GCA_030685595.1 | 1 |
| GCA_022648125.1 | 4 | GCA_030581795.1 | 1 | GCA_030685655.1 | 1 |
| GCA_022691445.1 | 6 | GCA_030584205.1 | 1 | GCA_030685675.1 | 1 |
| GCA_022699225.1 | 5 | GCA_030584225.1 | 1 | GCA_030685695.1 | 2 |
| GCA_022699545.1 | 2 | GCA_030584245.1 | 2 | GCA_030685715.1 | 2 |
| GCA_022699565.1 | 4 | GCA_030584265.1 | 8 | GCA_030685755.1 | 2 |
| GCA_022749455.1 | 5 | GCA_030584285.1 | 1 | GCA_030685775.1 | 1 |
| GCA_022811085.1 | 4 | GCA_030584305.1 | 1 | GCA_030685795.1 | 2 |
| GCA_022811125.1 | 2 | GCA_030584325.1 | 1 | GCA_030685835.1 | 1 |
| GCA_022811145.1 | 5 | GCA_030584345.1 | 1 | GCA_030685855.1 | 1 |
| GCA_022811165.1 | 6 | GCA_030584365.1 | 1 | GCA_030685875.1 | 1 |
| GCA_022811185.1 | 3 | GCA_030584385.1 | 2 | GCA_030685935.1 | 1 |
| GCA_022811205.1 | 4 | GCA_030584405.1 | 2 | GCA_030685975.1 | 2 |
| GCA_022811225.1 | 2 | GCA_030584425.1 | 1 | GCA_030685995.1 | 2 |
| GCA_022811245.1 | 2 | GCA_030584445.1 | 1 | GCA_030686015.1 | 1 |
| GCA_022811265.1 | 7 | GCA_030584465.1 | 1 | GCA_030686035.1 | 1 |
| GCA_022811285.1 | 3 | GCA_030584485.1 | 1 | GCA_030686075.1 | 1 |
| GCA_022811305.1 | 4 | GCA_030584505.1 | 1 | GCA_030686095.1 | 1 |
| GCA_022811325.1 | 1 | GCA_030584525.1 | 1 | GCA_030719015.1 | 3 |
| GCA_022811345.1 | 3 | GCA_030584545.1 | 1 | GCA_033130085.1 | 6 |
| GCA_022811365.1 | 2 | GCA_030584585.1 | 1 | GCA_033246985.1 | 3 |
| GCA_022811385.1 | 5 | GCA_030584605.1 | 1 | GCA_033247905.1 | 3 |
| GCA_022811405.1 | 6 | GCA_030584625.1 | 1 | GCA_033249005.1 | 3 |
| GCA_022811425.1 | 5 | GCA_030584645.1 | 2 | GCA_033249725.1 | 3 |
| GCA_022811445.1 | 5 | GCA_030584665.1 | 1 | GCA_033260105.1 | 2 |
| GCA_022811465.1 | 7 | GCA_030584685.1 | 1 | GCA_033802685.1 | 3 |
| GCA_023182815.2 | 3 | GCA_030644745.1 | 1 | GCA_034644175.1 | 2 |
| GCA_023182835.2 | 3 | GCA_030644765.1 | 1 | GCA_907163255.1 | 5 |
| GCA_023204955.1 | 8 | GCA_030644785.1 | 1 | GCA_907163315.1 | 5 |
| GCA_023204975.1 | 7 | GCA_030644805.1 | 1 | GCA_907165365.1 | 8 |
| GCA_023299325.1 | 5 | GCA_030644825.1 | 2 | GCA_907176135.1 | 9 |
| GCA_023299725.1 | 6 | GCA_030656695.1 | 1 | GCA_907176815.1 | 9 |
| GCA_023299745.1 | 4 | GCA_030656735.1 | 2 | GCA_907177285.1 | 9 |

**Supplementary Table S11. *Pediococcus pentosaceus* pan-genes mobileOG prediction results.**

| ID | RRR | P | T | STD | IE | ID | RRR | P | T | STD | IE |
| --- | --- | --- | --- | --- | --- | --- | --- | --- | --- | --- | --- |
| GCA_016652195.1 | 10 | 17 | 7 | 12 | 37 | GCA_025122155.1 | 9 | 12 | 1 | 5 | 21 |
| GCA_016726885.1 | 2 | 4 | 5 | 2 | 20 | GCA_025122175.1 | 3 | 3 | 7 | 1 | 12 |
| GCA_016921135.1 | 5 | 8 | 0 | 2 | 2 | GCA_025122195.1 | 7 | 8 | 9 | 5 | 21 |
| GCA_019008315.1 | 8 | 9 | 2 | 4 | 14 | GCA_025132875.1 | 3 | 5 | 0 | 1 | 7 |
| GCA_019614475.1 | 1 | 9 | 0 | 0 | 2 | GCA_025132915.1 | 6 | 10 | 1 | 6 | 22 |
| GCA_019793535.1 | 1 | 7 | 0 | 0 | 1 | GCA_025188055.1 | 1 | 11 | 0 | 0 | 1 |
| GCA_020882545.2 | 3 | 12 | 0 | 0 | 1 | GCA_025188065.1 | 1 | 3 | 0 | 0 | 0 |
| GCA_021124455.1 | 13 | 5 | 6 | 10 | 23 | GCA_025188105.1 | 5 | 3 | 4 | 4 | 24 |
| GCA_021378055.1 | 5 | 6 | 0 | 2 | 7 | GCA_025188125.1 | 3 | 6 | 0 | 0 | 1 |
| GCA_022394815.1 | 8 | 9 | 2 | 4 | 16 | GCA_025188145.1 | 8 | 2 | 3 | 4 | 18 |
| GCA_022483125.1 | 1 | 11 | 0 | 2 | 1 | GCA_025770455.1 | 10 | 13 | 5 | 5 | 21 |
| GCA_022483435.1 | 1 | 2 | 0 | 0 | 5 | GCA_025770465.1 | 7 | 6 | 3 | 4 | 17 |
| GCA_022483705.1 | 1 | 2 | 0 | 0 | 6 | GCA_025770495.1 | 8 | 13 | 14 | 2 | 14 |
| GCA_022484085.1 | 1 | 1 | 0 | 0 | 0 | GCA_026226215.1 | 1 | 4 | 0 | 0 | 1 |
| GCA_022641845.1 | 3 | 1 | 1 | 3 | 3 | GCA_027667435.1 | 4 | 5 | 0 | 2 | 14 |
| GCA_022641855.1 | 1 | 1 | 0 | 0 | 0 | GCA_027675905.1 | 2 | 4 | 3 | 2 | 10 |
| GCA_022642265.1 | 2 | 0 | 0 | 0 | 5 | GCA_027688855.1 | 5 | 5 | 8 | 0 | 16 |
| GCA_022642915.1 | 1 | 1 | 0 | 0 | 0 | GCA_027693945.1 | 4 | 6 | 1 | 2 | 15 |
| GCA_022643425.1 | 1 | 2 | 0 | 0 | 0 | GCA_028067445.1 | 8 | 9 | 2 | 4 | 14 |
| GCA_022644945.1 | 1 | 1 | 0 | 0 | 0 | GCA_028206435.1 | 14 | 22 | 12 | 9 | 29 |
| GCA_022645105.1 | 1 | 1 | 0 | 0 | 0 | GCA_028656215.1 | 1 | 7 | 0 | 0 | 1 |
| GCA_022645325.1 | 1 | 1 | 0 | 0 | 0 | GCA_028656295.1 | 4 | 10 | 3 | 2 | 23 |
| GCA_022646465.1 | 1 | 1 | 0 | 0 | 0 | GCA_028888655.1 | 1 | 5 | 0 | 0 | 1 |
| GCA_022646785.1 | 1 | 1 | 0 | 0 | 0 | GCA_028994255.1 | 10 | 9 | 5 | 2 | 21 |
| GCA_022646925.1 | 1 | 1 | 0 | 0 | 0 | GCA_029070885.1 | 9 | 9 | 13 | 6 | 19 |
| GCA_022678665.1 | 3 | 12 | 0 | 0 | 1 | GCA_029439595.1 | 8 | 15 | 3 | 3 | 18 |
| GCA_022690785.1 | 1 | 8 | 0 | 0 | 0 | GCA_029542265.1 | 2 | 9 | 3 | 2 | 5 |
| GCA_023277765.1 | 5 | 2 | 1 | 2 | 8 | GCA_029823395.1 | 7 | 2 | 3 | 4 | 17 |
| GCA_023277785.1 | 9 | 14 | 4 | 12 | 34 | GCA_030291815.1 | 3 | 13 | 0 | 1 | 2 |
| GCA_023277805.1 | 11 | 14 | 18 | 16 | 42 | GCA_030291835.1 | 3 | 1 | 1 | 2 | 7 |
| GCA_023369775.1 | 1 | 11 | 2 | 0 | 2 | GCA_030291855.1 | 1 | 4 | 0 | 0 | 4 |
| GCA_023507505.1 | 3 | 11 | 0 | 3 | 5 | GCA_030403405.1 | 12 | 8 | 11 | 7 | 23 |
| GCA_023740675.1 | 9 | 9 | 3 | 9 | 35 | GCA_030433975.1 | 3 | 7 | 5 | 3 | 12 |
| GCA_023743315.1 | 2 | 14 | 0 | 0 | 7 | GCA_030480445.1 | 1 | 2 | 0 | 0 | 0 |
| GCA_023743335.1 | 14 | 5 | 4 | 6 | 33 | GCA_030984705.1 | 5 | 11 | 0 | 4 | 2 |
| GCA_023744045.1 | 13 | 22 | 4 | 3 | 19 | GCA_033088855.1 | 7 | 7 | 0 | 2 | 6 |
| GCA_023744355.1 | 8 | 7 | 3 | 6 | 36 | GCA_033882205.1 | 7 | 5 | 3 | 0 | 20 |
| GCA_024349305.1 | 4 | 4 | 3 | 4 | 21 | GCA_034321905.1 | 4 | 3 | 6 | 2 | 22 |
| GCA_024539715.1 | 7 | 11 | 6 | 7 | 28 | GCA_035209715.1 | 2 | 5 | 3 | 2 | 10 |
| GCA_024580495.1 | 9 | 2 | 4 | 10 | 20 | GCA_035321745.1 | 3 | 8 | 0 | 5 | 18 |
| GCA_024580845.1 | 9 | 2 | 1 | 7 | 13 | GCA_910579815.1 | 2 | 8 | 0 | 0 | 12 |
| GCA_024622005.1 | 1 | 1 | 0 | 0 | 1 | GCA_925281455.1 | 2 | 4 | 5 | 2 | 10 |
| GCA_025122005.1 | 3 | 3 | 7 | 1 | 10 | GCA_925289295.1 | 3 | 4 | 3 | 4 | 24 |
| GCA_025122025.1 | 3 | 3 | 7 | 1 | 10 | GCA_925299425.1 | 4 | 4 | 3 | 4 | 24 |
| GCA_025122095.1 | 6 | 3 | 1 | 2 | 11 | GCA_938029495.1 | 8 | 8 | 13 | 4 | 31 |
| GCA_025122105.1 | 2 | 4 | 3 | 2 | 14 | GCA_949118755.1 | 2 | 4 | 0 | 0 | 5 |
| GCA_025122115.1 | 1 | 4 | 0 | 0 | 4 | GCA_949483205.1 | 1 | 4 | 0 | 0 | 0 |
| GCA_949483295.1 | 1 | 4 | 0 | 0 | 0 | R124 | 1 | 4 | 0 | 0 | 2 |

**Supplementary Table S12. *Pediococcus pentosaceus* pan-genes PlamidFinder prediction results.**

| ID | No. | ID | No. | ID | No. |
| --- | --- | --- | --- | --- | --- |
| GCA_016652195.1 | 1 | GCA_025132915.1 | 2 | GCA_030403405.1 | 1 |
| GCA_016921135.1 | 1 | GCA_025770465.1 | 1 | GCA_030984705.1 | 1 |
| GCA_023744045.1 | 2 | GCA_027667435.1 | 1 | GCA_033088855.1 | 1 |
| GCA_024539715.1 | 1 | GCA_027693945.1 | 1 | GCA_024580845.1 | 2 |
| GCA_024580495.1 | 1 | GCA_028206435.1 | 1 | GCA_030291835.1 | 1 |

**Supplementary Table S13. *Lactiplantibacillus plantarum* pan-genes mobileOG prediction results.**

| ID | RRR | P | T | STD | IE | ID | RRR | P | T | STD | IE |
| --- | --- | --- | --- | --- | --- | --- | --- | --- | --- | --- | --- |
| GCA_000466905.3 | 19 | 36 | 21 | 20 | 77 | GCA_026153115.1 | 10 | 26 | 5 | 7 | 22 |
| GCA_011170185.2 | 9 | 35 | 0 | 1 | 13 | GCA_026183415.1 | 10 | 18 | 3 | 4 | 22 |
| GCA_011304595.2 | 14 | 27 | 14 | 8 | 46 | GCA_026240755.1 | 10 | 26 | 4 | 2 | 20 |
| GCA_016598735.1 | 22 | 19 | 25 | 19 | 56 | GCA_026689375.1 | 15 | 32 | 12 | 13 | 70 |
| GCA_016775685.1 | 13 | 29 | 11 | 10 | 47 | GCA_026976315.1 | 13 | 23 | 6 | 7 | 26 |
| GCA_016812075.1 | 8 | 22 | 5 | 1 | 19 | GCA_027474465.1 | 16 | 22 | 15 | 11 | 55 |
| GCA_016838645.1 | 12 | 27 | 7 | 6 | 53 | GCA_027557615.1 | 6 | 20 | 0 | 1 | 9 |
| GCA_016894405.1 | 11 | 23 | 15 | 11 | 37 | GCA_027558615.1 | 8 | 16 | 4 | 1 | 15 |
| GCA_017068215.1 | 12 | 45 | 12 | 8 | 28 | GCA_027920405.1 | 9 | 28 | 7 | 3 | 56 |
| GCA_017068235.1 | 9 | 8 | 7 | 9 | 36 | GCA_028201575.1 | 13 | 41 | 14 | 6 | 41 |
| GCA_017301935.1 | 10 | 26 | 4 | 2 | 21 | GCA_028411375.1 | 4 | 17 | 6 | 6 | 9 |
| GCA_017351995.1 | 7 | 24 | 8 | 5 | 42 | GCA_028463965.1 | 14 | 37 | 5 | 11 | 40 |
| GCA_017576965.1 | 10 | 21 | 12 | 7 | 57 | GCA_028768485.1 | 5 | 20 | 0 | 0 | 7 |
| GCA_017742875.1 | 9 | 29 | 0 | 3 | 10 | GCA_028869445.1 | 15 | 28 | 10 | 8 | 21 |
| GCA_017798305.1 | 12 | 20 | 10 | 2 | 14 | GCA_029537295.1 | 21 | 28 | 20 | 22 | 55 |
| GCA_018351295.1 | 9 | 20 | 9 | 3 | 22 | GCA_029542245.1 | 9 | 30 | 0 | 3 | 10 |
| GCA_018588605.2 | 15 | 30 | 17 | 15 | 83 | GCA_029543005.1 | 7 | 28 | 4 | 4 | 25 |
| GCA_018588615.2 | 12 | 25 | 4 | 1 | 16 | GCA_029590535.1 | 13 | 10 | 3 | 7 | 28 |
| GCA_018588665.2 | 12 | 25 | 4 | 1 | 16 | GCA_029637825.1 | 22 | 31 | 19 | 9 | 48 |
| GCA_019076805.1 | 6 | 20 | 0 | 0 | 6 | GCA_029814785.1 | 6 | 20 | 0 | 1 | 9 |
| GCA_019211765.1 | 9 | 8 | 6 | 7 | 28 | GCA_029834415.1 | 6 | 20 | 0 | 1 | 9 |
| GCA_019211785.1 | 9 | 8 | 6 | 9 | 35 | GCA_029854235.1 | 8 | 30 | 3 | 2 | 21 |
| GCA_019321805.1 | 13 | 34 | 22 | 7 | 64 | GCA_029854315.1 | 10 | 25 | 6 | 5 | 36 |
| GCA_019399915.1 | 13 | 27 | 4 | 8 | 21 | GCA_029854335.1 | 14 | 39 | 11 | 12 | 74 |
| GCA_019425695.1 | 4 | 14 | 0 | 0 | 7 | GCA_029855105.1 | 13 | 49 | 3 | 5 | 20 |
| GCA_019469465.1 | 6 | 14 | 0 | 0 | 6 | GCA_029906425.1 | 11 | 19 | 22 | 7 | 29 |
| GCA_020881935.1 | 13 | 30 | 5 | 6 | 26 | GCA_030061915.1 | 9 | 24 | 1 | 3 | 14 |
| GCA_021279005.2 | 9 | 18 | 0 | 2 | 11 | GCA_030061935.1 | 13 | 18 | 17 | 10 | 59 |
| GCA_021559675.1 | 9 | 9 | 18 | 7 | 45 | GCA_030061955.1 | 6 | 34 | 6 | 3 | 18 |
| GCA_021559915.1 | 16 | 31 | 14 | 9 | 40 | GCA_030061985.1 | 12 | 19 | 9 | 11 | 60 |
| GCA_021560135.1 | 9 | 23 | 0 | 0 | 10 | GCA_030253605.1 | 8 | 32 | 3 | 3 | 28 |
| GCA_021650875.1 | 9 | 8 | 6 | 7 | 28 | GCA_030297695.1 | 9 | 27 | 1 | 1 | 11 |
| GCA_022558425.1 | 15 | 5 | 23 | 9 | 57 | GCA_030297715.1 | 7 | 22 | 17 | 8 | 29 |
| GCA_022810685.1 | 11 | 18 | 3 | 5 | 37 | GCA_030297735.1 | 5 | 22 | 0 | 0 | 7 |
| GCA_023207995.1 | 10 | 26 | 4 | 2 | 20 | GCA_030406025.1 | 8 | 24 | 0 | 1 | 10 |
| GCA_023278325.1 | 10 | 15 | 9 | 8 | 61 | GCA_030463605.1 | 10 | 24 | 13 | 2 | 18 |
| GCA_023347215.1 | 16 | 21 | 14 | 14 | 45 | GCA_030503695.1 | 13 | 32 | 17 | 6 | 60 |
| GCA_023348385.1 | 9 | 34 | 0 | 2 | 20 | GCA_030758995.1 | 12 | 19 | 14 | 6 | 53 |
| GCA_023348465.1 | 9 | 27 | 1 | 2 | 14 | GCA_031348585.1 | 7 | 9 | 1 | 0 | 6 |
| GCA_023348525.1 | 7 | 17 | 1 | 1 | 9 | GCA_031432955.1 | 10 | 18 | 7 | 3 | 21 |
| GCA_023370155.1 | 11 | 17 | 7 | 4 | 28 | GCA_031583165.1 | 14 | 16 | 7 | 13 | 60 |
| GCA_023973045.1 | 11 | 39 | 2 | 5 | 17 | GCA_031596315.1 | 8 | 14 | 3 | 2 | 29 |
| GCA_024137845.1 | 8 | 24 | 0 | 1 | 10 | GCA_031597175.1 | 13 | 24 | 4 | 5 | 32 |
| GCA_024137985.1 | 18 | 31 | 17 | 6 | 57 | GCA_031876755.1 | 4 | 2 | 0 | 0 | 25 |
| GCA_024181685.1 | 15 | 18 | 17 | 9 | 62 | GCA_031877725.1 | 10 | 19 | 0 | 0 | 10 |
| GCA_024181705.1 | 16 | 41 | 13 | 8 | 64 | GCA_032463565.1 | 9 | 28 | 5 | 7 | 32 |
| GCA_024396815.1 | 8 | 13 | 6 | 4 | 24 | GCA_032463585.1 | 9 | 28 | 5 | 7 | 33 |
| GCA_024442115.1 | 11 | 23 | 12 | 3 | 21 | GCA_032602185.1 | 11 | 18 | 4 | 4 | 22 |
| GCA_024732385.1 | 8 | 24 | 0 | 1 | 10 | GCA_032818175.1 | 10 | 24 | 13 | 2 | 19 |
| GCA_024758665.1 | 7 | 28 | 4 | 4 | 25 | GCA_032920365.1 | 7 | 18 | 6 | 3 | 36 |
| GCA_024758745.1 | 8 | 16 | 4 | 1 | 13 | GCA_032921125.1 | 10 | 27 | 5 | 3 | 30 |
| GCA_024800605.1 | 7 | 22 | 0 | 1 | 7 | GCA_033024555.1 | 10 | 28 | 5 | 6 | 25 |
| GCA_024969715.1 | 4 | 27 | 0 | 2 | 6 | GCA_033546835.1 | 6 | 22 | 1 | 4 | 9 |
| GCA_024969905.1 | 7 | 22 | 0 | 1 | 7 | GCA_033802745.1 | 9 | 16 | 2 | 1 | 25 |
| GCA_024970125.1 | 4 | 27 | 0 | 2 | 6 | GCA_033802805.1 | 6 | 27 | 0 | 2 | 8 |
| GCA_024970145.1 | 4 | 27 | 0 | 2 | 6 | GCA_034333585.1 | 13 | 22 | 5 | 4 | 45 |
| GCA_024970165.1 | 4 | 16 | 0 | 0 | 7 | GCA_034422915.1 | 13 | 22 | 18 | 12 | 49 |
| GCA_025144505.1 | 8 | 25 | 0 | 2 | 11 | GCA_034426955.1 | 13 | 17 | 20 | 11 | 55 |
| GCA_025402835.1 | 9 | 24 | 1 | 3 | 13 | GCA_034478365.1 | 14 | 22 | 18 | 13 | 49 |
| GCA_025723165.1 | 10 | 26 | 5 | 2 | 20 | GCA_034479655.1 | 11 | 32 | 14 | 3 | 24 |
| GCA_026013765.1 | 11 | 38 | 15 | 7 | 45 | GCA_035328725.1 | 4 | 17 | 0 | 0 | 11 |
| GCA_026016545.1 | 19 | 31 | 20 | 8 | 46 | GCA_035336205.1 | 13 | 37 | 4 | 6 | 23 |
| GCA_035338015.1 | 12 | 28 | 8 | 9 | 40 | Z108 | 8 | 24 | 0 | 2 | 13 |
| GCA_035588615.1 | 9 | 23 | 4 | 5 | 25 |  |  |  |  |  |  |

**Supplementary Table S14. *Lactiplantibacillus plantarum* pan-genes PlamidFinder prediction results.**

| ID | No. | ID | No. | ID | No. |
| --- | --- | --- | --- | --- | --- |
| Z108 | 1 | GCA_023973045.1 | 5 | GCA_029854235.1 | 3 |
| GCA_000466905.3 | 2 | GCA_024137845.1 | 1 | GCA_029854315.1 | 5 |
| GCA_011170185.2 | 8 | GCA_024137985.1 | 5 | GCA_029854335.1 | 4 |
| GCA_016598735.1 | 4 | GCA_024181685.1 | 9 | GCA_029855105.1 | 6 |
| GCA_016775685.1 | 5 | GCA_024181705.1 | 7 | GCA_029906425.1 | 6 |
| GCA_016812075.1 | 11 | GCA_024396815.1 | 1 | GCA_030061915.1 | 6 |
| GCA_016838645.1 | 3 | GCA_024442115.1 | 6 | GCA_030061935.1 | 8 |
| GCA_016894405.1 | 8 | GCA_024732385.1 | 1 | GCA_030061955.1 | 1 |
| GCA_017068215.1 | 6 | GCA_024758665.1 | 13 | GCA_030061985.1 | 8 |
| GCA_017068235.1 | 6 | GCA_024758745.1 | 5 | GCA_030253605.1 | 5 |
| GCA_017301935.1 | 6 | GCA_024800605.1 | 5 | GCA_030297695.1 | 7 |
| GCA_017351995.1 | 9 | GCA_024969715.1 | 5 | GCA_030297715.1 | 10 |
| GCA_017576965.1 | 10 | GCA_024969905.1 | 5 | GCA_030297735.1 | 5 |
| GCA_017742875.1 | 4 | GCA_024970125.1 | 5 | GCA_030406025.1 | 1 |
| GCA_017798305.1 | 5 | GCA_024970145.1 | 5 | GCA_030463605.1 | 5 |
| GCA_018351295.1 | 3 | GCA_024970165.1 | 1 | GCA_030503695.1 | 3 |
| GCA_018588605.2 | 3 | GCA_025144505.1 | 4 | GCA_030758995.1 | 6 |
| GCA_018588615.2 | 3 | GCA_025402835.1 | 6 | GCA_031432955.1 | 3 |
| GCA_018588665.2 | 3 | GCA_025723165.1 | 6 | GCA_031583165.1 | 6 |
| GCA_019076805.1 | 6 | GCA_026013765.1 | 3 | GCA_031596315.1 | 5 |
| GCA_019211765.1 | 6 | GCA_026016545.1 | 3 | GCA_031597175.1 | 7 |
| GCA_019211785.1 | 6 | GCA_026153115.1 | 1 | GCA_031876755.1 | 13 |
| GCA_019321805.1 | 13 | GCA_026183415.1 | 8 | GCA_031877725.1 | 15 |
| GCA_019399915.1 | 7 | GCA_026240755.1 | 6 | GCA_032463565.1 | 7 |
| GCA_019425695.1 | 1 | GCA_026689375.1 | 3 | GCA_032463585.1 | 7 |
| GCA_019469465.1 | 5 | GCA_026976315.1 | 2 | GCA_032602185.1 | 8 |
| GCA_020881935.1 | 3 | GCA_027474465.1 | 12 | GCA_032818175.1 | 5 |
| GCA_021279005.2 | 8 | GCA_027557615.1 | 7 | GCA_032920365.1 | 4 |
| GCA_021559675.1 | 6 | GCA_027558615.1 | 5 | GCA_032921125.1 | 3 |
| GCA_021559915.1 | 2 | GCA_027920405.1 | 1 | GCA_033024555.1 | 3 |
| GCA_021560135.1 | 1 | GCA_028201575.1 | 4 | GCA_033546835.1 | 7 |
| GCA_021650875.1 | 6 | GCA_028463965.1 | 1 | GCA_033802745.1 | 3 |
| GCA_022558425.1 | 1 | GCA_028768485.1 | 1 | GCA_033802805.1 | 11 |
| GCA_022810685.1 | 13 | GCA_028869445.1 | 7 | GCA_034333585.1 | 9 |
| GCA_023207995.1 | 6 | GCA_029537295.1 | 4 | GCA_034422915.1 | 2 |
| GCA_023278325.1 | 7 | GCA_029542245.1 | 3 | GCA_034426955.1 | 6 |
| GCA_023347215.1 | 3 | GCA_029543005.1 | 12 | GCA_034478365.1 | 2 |
| GCA_023348385.1 | 6 | GCA_029590535.1 | 3 | GCA_034479655.1 | 5 |
| GCA_023348465.1 | 3 | GCA_029637825.1 | 3 | GCA_035328725.1 | 7 |
| GCA_023348525.1 | 9 | GCA_029814785.1 | 7 | GCA_035336205.1 | 1 |
| GCA_023370155.1 | 7 | GCA_029834415.1 | 7 | GCA_035338015.1 | 7 |
| GCA_035588615.1 | 1 |  |  |  |  |

**Supplementary Table S15. ANI values between the isolates and their closest type strains.**

| Isolate | Species | Reference Genome (Accession) | ANI (%) |
| --- | --- | --- | --- |
| F130 | *Enterococcus faecium* | GCA_029023785.1 | 98.97 |
| R124 | *Pediococcus pentosaceus* | GCA_001437285.1 | 98.77 |
| Z108 | *Lactiplantibacillus plantarum* | GCA_000143745.1 | 99.04 |
